# Does the Type of Semen Affect the Phosphoproteome of Turkey (*Meleagris gallopavo*) Spermatozoa?

**DOI:** 10.3390/ijms26083467

**Published:** 2025-04-08

**Authors:** Katarzyna T. Rafalska, Aleksandra Orzołek, Joanna Ner-Kluza, Paweł Wysocki

**Affiliations:** 1 Department of Animal Biochemistry and Biotechnology, Faculty of Animal Bioengineering, University of Warmia and Mazury in Olsztyn, Oczapowskiego 5, 10-719 Olsztyn, Poland; kasia.rafalska13@gmail.com (K.T.R.); pawel.wysocki@uwm.edu.pl (P.W.); 2 Department of Biochemistry and Neurobiology, Faculty of Materials Science and Ceramics, AGH University, Mickiewicza 30, 30-059 Cracow, Poland; nerjoanna@gmail.com

**Keywords:** turkey, spermatozoa, YSS, viability, antioxidant status, sperm phosphoproteome

## Abstract

Yellow semen syndrome (YSS) is an increasingly common reproductive health problem in male turkeys. This condition is characterised by a yellow discolouration of semen, often linked to decreased semen quality and fertility. Yellow semen syndrome poses a significant concern due to its negative impact on the reproductive performance of turkeys. Phosphorylation is one of the major post-translational modifications of proteins. A better understanding of the function of the sperm phosphoproteome is crucial for the advancement of reproductive biology and the development of therapies for male infertility. Spermatozoa from semen samples with YSS were characterised by lower levels of malondialdehyde (MDA), reduced plasma membrane integrity (PMI), and decreased mitochondrial membrane potential (MMP). However, these samples showed increased antioxidant enzyme activity and an elevated glutathione (GSH) content. Yellow sperm also had a lower percentage of viable cells and a higher proportion of apoptotic and necrotic cells. The phosphoproteins identified in turkey sperm play key roles in sperm maturation, the development of a functional motility apparatus, efficient cellular metabolism, protection against oxidative stress, and successful fertilisation of an egg. Yellow semen syndrome altered the phosphorylation of turkey sperm proteins on serine, threonine (*p* ≤ 0.05), and tyrosine residues, which could have influenced the metabolism and physiology of spermatozoa in yellow semen samples, thus affecting their reproductive potential. These findings highlight the impact of YSS on sperm function, including phosphorylation-dependent processes that are crucial for reproduction.

## 1. Introduction

Yellow semen syndrome (YSS) is endemic in the turkey population, affecting an estimated 10–16% of males. While the exact cause of YSS remains unknown, potential factors include genetic predisposition, suboptimal photoperiods, and close inbreeding [1]. Yellow semen is considered to be of poor quality because it contains abnormal sperm cells, macrophages, bacteria, and numerous proteins from the seminal plasma. The yellow discolouration is typically caused by abnormal proteins, lipids, or other substances that may affect overall reproductive efficiency [1,2,3,4,5,6,7]. Yellow semen syndrome impacts spermatozoa by decreasing motility, impairing viability, altering morphology, and inducing oxidative stress. In turkeys affected by YSS, the ejaculate volume and sperm concentration typically remain constant, but the number of abnormal spermatozoa, spermatids, and microbes increases [1,8]. In addition to colour differences, YSS is associated with elevated levels of seminal plasma proteins and cholesterol, as well as increased activity of aspartate aminotransferase, acid phosphatase, and superoxide dismutase [3,9]. Słowińska et al. [5] identified distinct differences in the proteomic profiles of white and yellow seminal plasma. In particular, the expression of transthyretin, serum albumin-like protein, hemopexin-like protein, and immunoglobulin light chain V-J-C was found to be three times higher in YSS-affected semen. Słowińska et al. [6] also demonstrated that differentially expressed metabolites in white and yellow seminal plasma play key roles in sperm physiology, including carbohydrate and lipid metabolism, cell signalling, and cellular development.

Phosphorylation and dephosphorylation are dynamic processes. Evidence suggests that proper sperm maturation in the epididymis also depends on the regulation of protein phosphorylation and dephosphorylation events within spermatozoa. Phosphorylation regulators play a key role in signal transduction by modulating the activity of substrate proteins, either activating or inhibiting their function. Phosphorylation-based post-translational modification enables eukaryotic cells and spermatozoa to dynamically regulate intracellular signalling and physiological states [10]. The phosphorylation of sperm proteins is critical for male fertility, as this post-translational modification is essential at every stage of sperm cell development. Phosphorylation is vital for sperm differentiation, maturation, and functionality [11]. In addition, increased tyrosine phosphorylation of sperm proteins is critical for regulating sperm motility [12] and capacitation [13]. The level of tyrosine phosphorylation in human sperm is strongly correlated with sperm-zona binding capacity, and changes in tyrosine phosphorylation have been observed in subfertile individuals, highlighting its physiological role in fertilisation [14,15]. Despite significant technological advances, only a limited number of phosphoproteomics studies have investigated male infertility. New research findings will contribute to a better understanding of the effects of protein phosphorylation on sperm physiology, thus providing valuable insights into male fertility [10].

Therefore, the aim of this study was to analyse and characterise the turkey sperm phosphoproteome, focusing on the prevalence of YSS in male turkeys.

## 2. Results

### 2.1. Biological Parameters of White and Yellow Turkey Sperm

Significant (*p* ≤ 0.05) and highly significant (*p* ≤ 0.001) differences were observed in the parameters characterising spermatozoa from white and yellow semen. The kinetic properties of sperm motility, such as TMOT, VCL, and BCF, were higher in white semen, whereas VSL, STR, and LIN were higher in yellow semen. Sperm concentration, PMI in the acrosomal region, sperm mitochondrial function, and the percentage of viable sperm were significantly higher in white semen. In contrast, the percentages of apoptotic and necrotic sperm were significantly higher in yellow semen. SOD and GPx activity, along with the GSH content, were considerably higher in spermatozoa derived from YSS ejaculates (Table 1).

### 2.2. Correlations Between Selected Parameters of Spermatozoa Derived from White and Yellow Semen

The relationships between motility or viability and the antioxidant parameters of spermatozoa derived from white and yellow semen were evaluated by calculating correlation coefficients. In white semen, no correlations were observed between sperm antioxidant status and motility (Table 2). In turn, strong positive correlations between CAT activity and sperm motility were noted in yellow semen. In addition, motility parameters were bound by positive correlations with GSH content and negative correlations with MDA levels (Table 3). The relationship between sperm concentration and the activity of antioxidant enzymes exerted both negative and positive effects on MDA levels (Table 4 and Table 5), although to a much lesser extent in yellow semen. A positive correlation was noted between SOD activity and the percentage of NO-producing sperm in white semen (Table 4), as well as between CAT activity and the percentage of sperm exhibiting pro-apoptotic changes in yellow semen (Table 5). Interestingly, the correlations between GSH content, MDA levels, and the antioxidant status of sperm were characterised by opposite signs in white and yellow semen.

### 2.3. Isolation and Identification of Sperm Phosphoproteins from White and Yellow Semen

**Figure 1:**
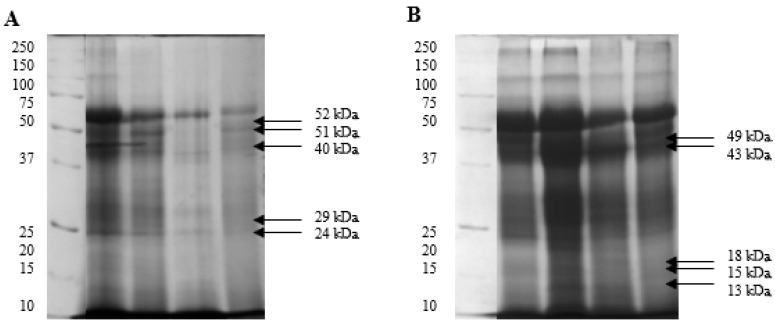
Phosphoprotein profiles of spermatozoa identified by SDS-PAGE in white (**A**) and yellow (**B**) turkey semen.

In the phosphoprotein profiles of spermatozoa, only the 58 kDa protein was present in the proteome of each individual. The proteins characteristic of white semen had a molecular weight of 52, 51, 40, 29, and 24 kDa, whereas the proteins characteristic of yellow semen had a molecular weight of 49, 43, 18, 15, and 13 kDa (Figure 1). The OD of protein fractions confirmed these differences in each type of semen (Table 6). The sperm phosphoproteins identified by nano LC-MS/MS mass spectrometry are presented in the Supplementary Materials (Table S1).

### 2.4. Immunodetection of Sperm Phosphoproteins in White and Yellow Semen

**Figure 2:**
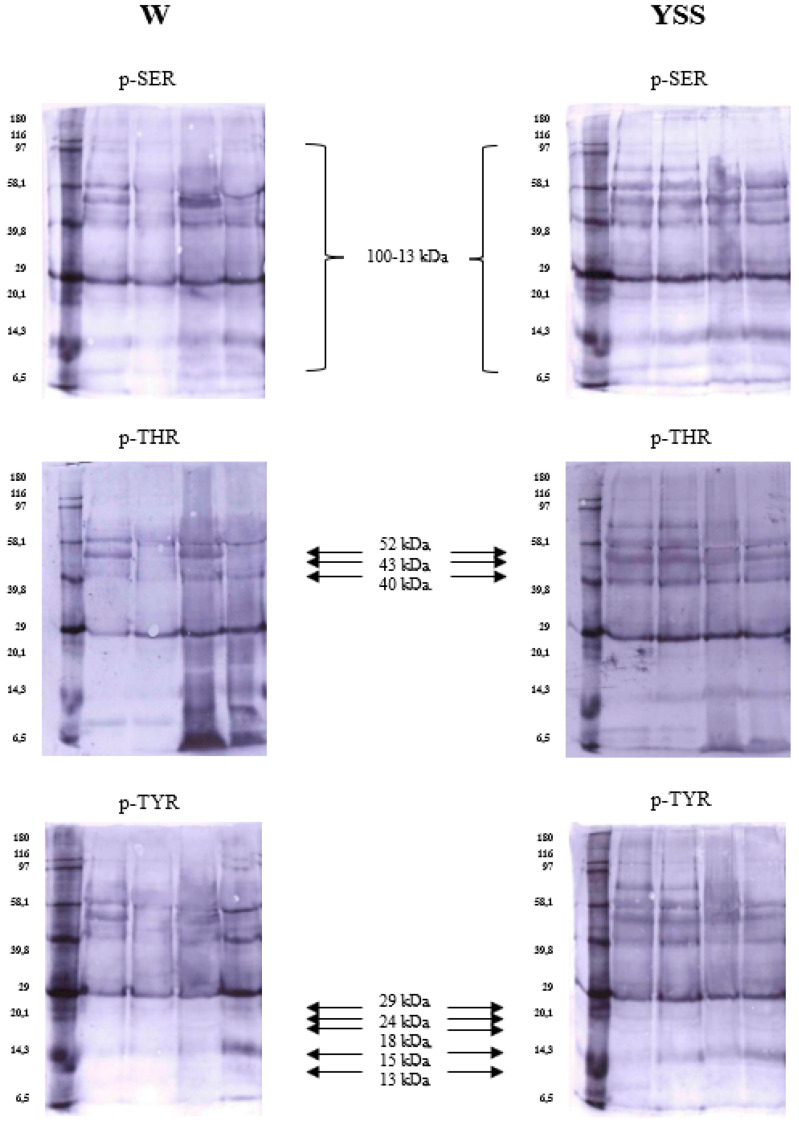
Immunodetection of protein fractions from white (W) and yellow (YSS) spermatozoa phosphorylated on serine (pSER), threonine (pTHR), and tyrosine (pTYR) residues.

Increased protein phosphorylation was observed in yellow sperm. Proteins with a molecular weight of 100 to 13 kDa underwent the strongest phosphorylation (Figure 2). Proteins with a molecular weight of 52, 51, 49, 43, 40, 29, 24, 18, 15, and 13 kDa had different phosphorylation profiles in white and yellow semen (Figure 2). Proteins with an average molecular weight were more strongly phosphorylated, especially in yellow ejaculates. Interestingly, phosphorylation on serine, threonine, and tyrosine residues was equally increased in this type of semen. An analysis of OD values in selected sperm fractions from yellow semen revealed that proteins with a molecular weight of 52, 43, and 40 kDa were more readily phosphorylated on phosphothreonine residues (Table 7). However, no significant differences in protein phosphorylation on serine and tyrosine residues were found between white and yellow semen.

**Figure 3:**
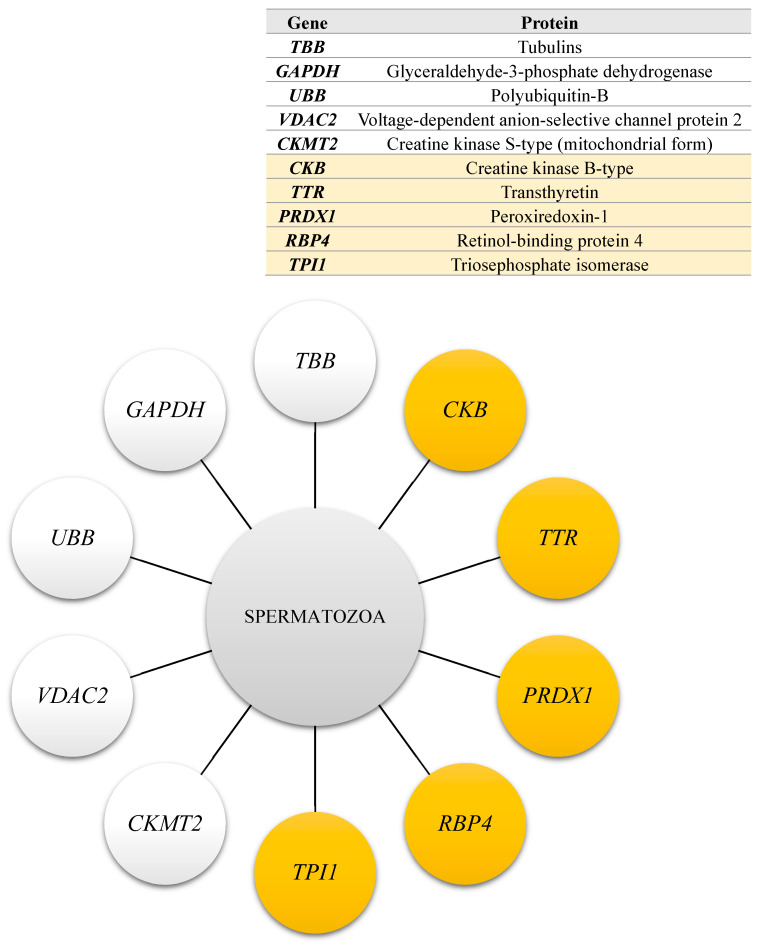
Genes encoding proteins that underwent differential phosphorylation in spermatozoa derived from white (white circles) and yellow (yellow circles) semen.

The genes encoding proteins that underwent differential phosphorylation in spermatozoa derived from white and yellow turkey semen are presented in Figure 3.

### 2.5. The Results of Functional Analysis of the Identified Sperm Phosphoproteins

**Figure 4:**
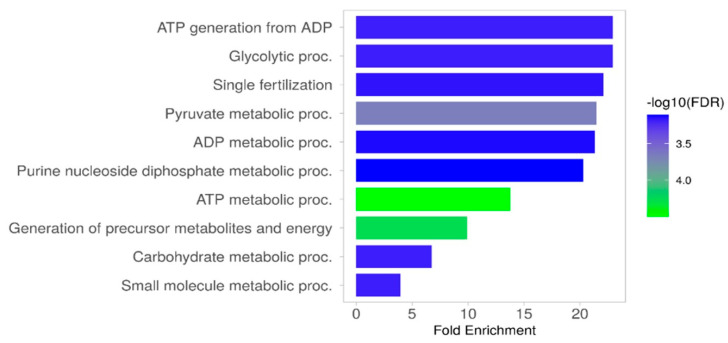
The key functions of the identified turkey sperm phosphoproteins. Source: own elaboration based on the GO Biological Process database in ShinyGO 0.77.

The analysis, performed using the ShinyGo 0.77 programme (setting: species—*Gallus gallus*, database—GO Biological Process), revealed that sperm phosphoproteins play key roles in energy generation in cells, processes associated with glucose and pyruvate metabolism, interactions between and binding of spermatozoa to egg cells, fertilisation, metabolic processes involving nucleosides, nucleotides, and low-molecular-weight compounds (Figure 4).

**Figure 5:**
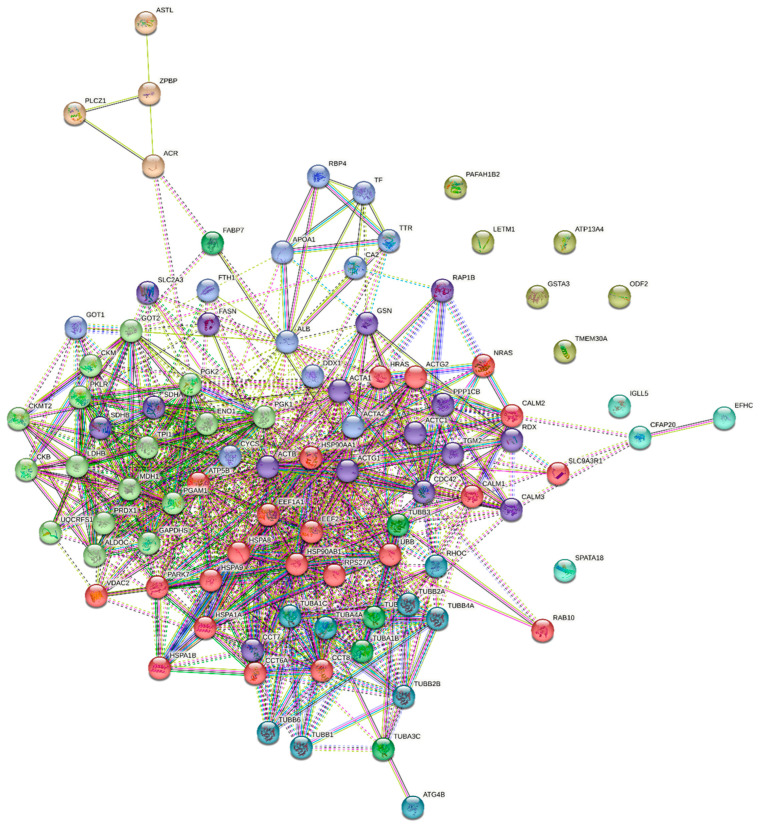
Network of protein–protein interactions in the phosphoproteins identified in turkey sperm based on the STRING 11.5 database. The proteins were divided into 11 functional groups. The lines connecting individual proteins represent the interactions. The types of interactions are marked with differently coloured lines: turquoise—known interactions that were identified in selected databases; pink—known interactions that were determined experimentally; green—interactions predicted based on gene neighbourhood; red—interactions that are likely on the assumption that genes are fused; blue—interactions predicted based on the co-occurrence of genes in metabolic pathways; yellow—data from the database; black—gene co-expression; purple—protein homology. Dashed lines represent the interactions between proteins from different groups.

In turn, an analysis of protein–protein interactions revealed a functional proteomic network in turkey spermatozoa (Figure 5). The network was divided into nine groups (clusters) based on the probability of interactions between proteins in each group. The first cluster included proteins encoded by the following genes: *ACTA1*, *ACTB*, *ACTC1*, *ACTG1*, *CALM3*, *CCT7*, *CDC42*, *FASN*, *GSN*, *PPP1CB*, *RAP1B*, *RDX*, *SDHA*, *SDHB*, *SLC2A3*, and *TGM2*. The identified proteins had different functions, including succinate metabolism, formation of actin filaments, and localisation of proteins in cells. The interactions between actin and radixin received a score of 0.485 to 0.998. The *ACTB* gene interacted with all proteins in the complex, and gelsolin (*GSN*) interacted with *ACTA1* (0.998), *ACTB* (0.874), *ACTC1* (0.810), and *ACTG1* (0.981). The second group consisted of proteins encoded by the following genes: *ACTA2*, *ALB*, *APOA1*, *CA2*, *CYCS*, *DDX1*, *FTH1*, *GOT1*, *RBP4*, *TF*, and *TTR*. These proteins have diverse functions. They are involved in HDL particle remodelling, retinoid transport and metabolism, iron absorption and transport, and post-translational protein phosphorylation. *ALB* entered into the strongest interactions with *APOA1* and cytochrome C (*CYCS*) (0.860). One of the proteins, the ATP-dependent RNA helicase (*DDX1*), did not interact with other proteins in this group; however, it did interact with other polypeptides within the sperm proteomic network. The third cluster comprised polypeptides encoded by the following genes: *ALDOC*, *CKB*, *CKM*, *CKMT2*, *ENO1*, *GAPDHS*, *GOT2*, *LDHB*, *MDH1*, *PGAM1*, *PGK1*, *PGK2*, *PKLR*, *PRDX1*, *TPI1*, and *UQCRFS1*. Most of these peptides are enzymes involved in sperm metabolism, including glycolysis, gluconeogenesis, phosphocreatine and ATP synthesis, as well as processes associated with NAD/NADH metabolism. The strongest interactions were observed between *TPI1* and *PGK1* (0.999), *GAPDH* (0.997), *PGK2* (0.996), *ENO1* (0.996), *ALDOC* (0.982), *GOT2* (0.971), and *PKLR* (0.956). A strong interaction was also found between *TPI1* and the Rieske subunit of the cytochrome b-c1 complex (*UQCRFS1*) (0.703), and between peroxiredoxin 1 (*PRDX1*) and *ENO1* (0.704). The following genes encoded the fourth group of proteins: *FABP7*, *TUBA1A*, *TUBA1B*, *TUBA3C*, and *TUBB3*. These proteins are an integral part of the sperm cytoskeleton, and they exhibit GTPase activity and bind GTP. The strongest interactions were observed between the beta 3 chain of tubulin (*TUBB3*) and tubulin chains *TUBA1A* (0.993) and *TUBA1B* (0.997). The interaction between *TUBB3* and fatty acid-binding protein 7 (*FABP7*) (0.544) was also relatively strong. The fifth cluster contained polypeptides encoded by the following genes: *ACR*, *ASTL*, *PLCZ1*, and *ZPBP*. All of these proteins are responsible for oocyte activation, oocyte–sperm binding, and fertilisation processes. The identified proteins entered into moderately strong interactions (0.426–0.575). The following genes encoded the proteins in the sixth group: *CFAP20*, *EFHC2*, *IGLL5*, and *SPATA18*. In this group, only a single interaction was noted between *CFAP20* and *EFHC2*, with a mean confidence interval (0.470). The seventh cluster consisted of polypeptides encoded by the following genes: *ATP13A4*, *GSTA3*, *LETM1*, *ODF2*, *PAFAH1B2*, and *TMEM30A*. None of these proteins entered into interactions. The eighth group of proteins was encoded by the following genes: *ATG4B*, *RHOC*, *TUBA1C*, *TUBA4A*, *TUBB1*, *TUBB2A*, *TUBB2B*, *TUBB4A*, and *TUBB6*. All of these proteins are involved in the organisation of the cell cytoskeleton. The last cluster included proteins encoded by *ACTG2*, *ATP5B*, *CALM1*, *CALM2*, *CCT6A*, *CCT8*, *EEF1A1*, *EEF2*, *HRAS*, *HSP90AA1*, *HSP90AB1*, *HSPA1A*, *HSPA1B*, *HSPA8*, *HSPA9*, *NRAS*, *PARK7*, *RAB10*, *RPS27A*, *SLC9A3R1*, *UBB*, and *VDAC2* genes. The polypeptides encoded by these genes are responsible for cellular adaptation to heat and oxidative stress, modulation of kinase activity, phosphoprotein phosphatase and telomerase, protein folding and stabilisation, cell differentiation, regulation of the apoptotic signalling pathway and biological cell quality, DNA damage detection, signal transduction, and intracellular transport. The main protein in this group was *HSP90AA1*, which entered into the highest number of strong interactions with other proteins (0.431–0.999). Heat shock proteins (HSPs) interacted mainly with other members of this family.

## 3. Discussion

Proteins play a crucial role in sperm function by influencing reproductive processes such as sperm transport within the female reproductive tract and sperm–egg interactions [16]. For example, the spermatozoa of the most fertile chickens have higher levels of metabolic and antioxidant enzymes. In contrast, the spermatozoa of less fertile males have a higher proportion of structural proteins [17]. Variations in the quality and quantity of these proteins can disrupt biological functions.

### 3.1. The Differences in Biological Parameters of White and Yellow Turkey Sperm

The present study demonstrated that the type of turkey semen has a significant effect on sperm parameters. The values of TMOT, VCL, and BCF were markedly higher in white semen. In contrast, the values of VSL, STR, and LIN were considerably higher in yellow semen. Sperm with higher VCL values have less fragmented DNA and, consequently, higher fertilisation potential [18]. Domoslawska et al. [19] reported that the parameters VAP, VCL, VSL, and BCF are good indicators of the fertilisation potential of canine spermatozoa because they denote their ability to move progressively and penetrate the zona pellucida [20]. Moreover, in vitro tests revealed that higher values of VCL and BCF were also associated with more efficient and faster oocyte penetration [21]. According to Pilane and Mapeka [22], elevated values of STR and LIN are indicative of oxidative stress in the semen of roosters. They are positively correlated with the percentage of sperm with fragmented DNA [23]. Given the above, the results of the present study suggest that spermatozoa from yellow semen are characterised by lower quality than spermatozoa from white semen and that yellow semen is more susceptible to oxidative stress.

Spermatozoa contain antioxidant enzymes. In general, SOD activity is higher in spermatozoa than in plasma, but this trait is species-specific. Turkey spermatozoa are characterised by lower SOD activity than the spermatozoa of roosters, drakes, shrikes, and quail [24]. Furthermore, GPx activity is also lower in the sperm of turkeys than in other poultry species. In turkeys, GPx activity is lower in sperm cells than in seminal plasma [25]. Several studies have shown that specific antioxidants protect the sperm plasma membrane from damage, thereby contributing to sperm survival [26]. Słowińska et al. [9] demonstrated that SOD activity, a key component of the antioxidant defence system in semen, was higher in birds with YSS. In contrast, Rafalska et al. [27] found no differences in SOD activity between white and yellow seminal plasma. However, GPx activity was significantly lower in white semen, while CAT activity was comparable in both types of semen. In the current study, yellow semen was characterised by a lower sperm concentration, and lower PMI and mitochondrial function, but higher activity of selected antioxidant enzymes such as SOD, GPx, and CAT. Similar observations were made by Koziorowska-Gilun et al. [28] who found that CAT activity was four times higher and SOD activity was nearly twice as high in spermatozoa from yellow than white turkey semen. The cited authors also reported that the mitochondrial function of sperm from white turkey semen was bound by a positive (*p* ≤ 0.05) correlation with SOD activity and a negative correlation with CAT activity. These correlations were not fully corroborated by the results of this study, where SOD activity was positively correlated with sperm MMP, but the correlation was not statistically significant. In the present study, SOD and CAT activity was bound by a negative correlation with sperm concentration in white semen. Furthermore, a strong correlation was noted between sperm concentration and MDA levels, providing indirect evidence that an increase in sperm concentration accelerates the peroxidation of plasma membrane lipids. Kobayashi et al. [29] suggested that SOD activity in human sperm is significantly correlated with the percentage of mobile gametes. This observation was not confirmed in the present study, where sperm motility was correlated with CAT activity only in yellow semen. Positive correlations were found between CAT activity, GPx activity, and PMI in spermatozoa derived from yellow semen [28]. In the present study, CAT activity was correlated with sperm viability. Koziorowska et al. [28] suggested that the reduced quality of sperm from yellow semen may be associated with lower values of PMI and MMP. This observation was confirmed in the present study, which also revealed an increased activity of antioxidant enzymes in sperm. In addition, the GSH content was significantly higher in spermatozoa from individuals with YSS. Excessive accumulation of GSH often leads to reductive stress, redox imbalance, and cytotoxicity. Interestingly, an increase in mitochondrial GSH levels affects the electron transport chain, promotes excessive generation of superoxide anion radicals, and increases SOD activity in sperm [30]. Glutathione may be essential for preserving the mobility of turkey gametes; however, it can also compromise sperm survival, as indicated by the correlation coefficients determined in white and yellow turkey semen. Nevertheless, the increased antioxidant protection of spermatozoa from yellow semen, as manifested by the increase in GSH levels, may be due to an attempt to maintain the functionality of sperm mitochondria. This finding is supported by the results of our previous study [27] where seminal plasma GSH and MDA levels were significantly elevated in birds with YSS, suggesting that sperm cells in yellow semen may require increased antioxidant protection. Mitochondria generate energy that is essential for sperm motility. The production of reactive oxygen species (ROS) increases significantly in damaged or structurally compromised mitochondria, which can impair mitochondrial function [31]. The enzymes regulating tyrosine phosphorylation and dephosphorylation in sperm proteins may be targeted by ROS, as mild oxidative conditions enhance protein tyrosine phosphorylation and trigger the acrosome reaction [32]. Gomez et al. [33] reported that ROS levels are inversely proportional to sperm quality in fresh ejaculates. Research has also shown that the presence of lipid peroxidation products in semen significantly contributes to a decline in sperm motility [34].

### 3.2. The Role of Protein Phosphorylation/Dephosphorylation Processes in Turkey Spermatozoa

The mechanisms that regulate phosphorylation and dephosphorylation processes in sperm cells should be identified and elucidated to expand our understanding of reproductive function in turkeys [10]. The development of sperm structures and the achievement of motility are controlled by a tightly coordinated phosphorylation process of selected ejaculate proteins. The phosphorylation of sperm proteins determines spermatogenesis, along with the acquisition of motility and capacitation, and it ultimately affects ejaculate quality. Studies investigating the sperm phosphoproteome in various animal species have shown that phosphorylation also influences sperm formation and maturation [10]. The phosphorylation of sperm proteins on tyrosine residues affects sperm capacitation and egg fertilisation. Although spermatozoa from ejaculates of varied quality differ in their fertilising capacity, the relationship between the phosphorylation of proteins on tyrosyl residues and their functionality and quality remains unclear [35]. Phosphorylated serine and threonine residues not only regulate sperm motility but are also involved in cellular processes such as proliferation, cell differentiation, and protein folding [36]. In turkey spermatozoa, phosphorylated residues, mainly serine and tyrosine residues, may be associated with the activity of specific proteins. These changes may be physiologically important in preparing phosphorylated proteins for specific functions. In somatic cells, glycogen synthase kinase 3 (GSK-3) is activated when it is phosphorylated on tyrosine and serine residues [37,38]. The phosphorylation of GSK-3 on serine residues increases significantly when sperm pass through the epididymis, and it substantially affects the achievement of motility. The expression of serine phosphoproteins is higher in spermatozoa originating from the cauda epididymis than in those originating from the head of the epididymis. However, the molecular weight of GSK-3α was higher in sperm originating from the cauda epididymis than in sperm from the head of the epididymis. There is evidence to suggest that GSK-3α may be phosphorylated on either serine or tyrosine residues in spermatozoa derived from the head of the epididymis, but it may be phosphorylated on both residues in spermatozoa originating from the cauda epididymis [39]. This observation suggests that sperm proteins in white turkey semen must be phosphorylated on both types of amino acid residues to become active. Several phosphoproteins associated with the acquisition of sperm motility, including axoneme proteins, have been identified to date. Spermatozoa with low fertilising ability were characterised by hyperactivated motility, and capacitation and acrosome reactions proceeded rapidly in these cells due to disruptions in the phosphorylation of sperm proteins [40]. According to the literature, the extent to which various proteins undergo phosphorylation differs in spermatozoa with high and low motility. In the current study, electrophoresis supported the identification of 12 polypeptides with multiple degrees of phosphorylation, which were correlated with changes in sperm motility. Protein phosphorylation increases during the capacitation process, and it determines fertilisation success in many species. Hyperactivated motility enables sperm to penetrate the ovarian thalamus and the zona pellucida, and it is associated mainly with the phosphorylation of tyrosine residues in the sperm head. Thus, the achievement of a tyrosine phosphorylation threshold in spermatozoa may be related to hyperactivated motility [41]. In the present study, numerous sperm proteins underwent phosphorylation. Most of them were phosphorylated on serine, threonine, and tyrosine residues. In mice, phosphorylation can be triggered by proline [36]. In turn, in boar spermatozoa, proteins are phosphorylated on serine and threonine residues mainly at an early stage of capacitation. This process is catalysed by protein kinase A (PKA), which indicates that serine/threonine kinases are activated before tyrosine phosphorylation [42]. Interestingly, turkey spermatozoa were characterised by similar phosphorylation profiles of serine and tyrosine residues. This suggests that proteins may need to attach phosphate groups to both serine and tyrosine residues to achieve full biological activity.

### 3.3. The Role of Selected Sperm Phosphoproteins in Turkey Spermatozoa

In the current study, a total of 92 phosphoproteins were identified in turkey spermatozoa. Sperm phosphoproteins are involved in ATP generation, carbohydrate metabolism, and fertilisation. Phosphoproteome analysis (HPLC-MS/MS triple TOF) comparing spermatozoa with varying motility revealed 210 phosphopeptides corresponding to 119 proteins. The analysis also demonstrated that in low-motility spermatozoa, 40% of phosphoproteins were involved in metabolic processes (catabolism, protein transport, lipid biosynthesis), 25% participated in spermatogenesis and sperm function, 8% were involved in immune responses, and 6% were responsible for DNA repair. In contrast, in high-motility spermatozoa, 48% of phosphoproteins were associated with spermatogenesis and sperm function (mainly motility), while only 8% were involved in metabolic processes [43]. The present study demonstrated that the primary role of phosphoproteins identified in turkey spermatozoa is to generate ATP, thereby maintaining adequate levels of sperm motility necessary to traverse the female reproductive tract and fertilise the egg. Consequently, the presence of numerous proteins involved in sperm maturation, the elimination of abnormal gametes, and the development of the motility apparatus in turkey semen underscores the importance of motile sperm with a normal morphology. A phosphorylated form of phospholipase C zeta 1 (PLCZ1) was identified in turkey spermatozoa. The PLC family of proteins is a group of enzymes that catabolise phosphatidylinositol-4,5-bisphosphate to IP3 and diacylglycerol (DAG). Thirteen isoforms of PLC have been identified based on their function and protein structure. Phospholipase C zeta 1 is the least known isoform, with a molecular weight of 70–75 kDa in various species [44]. Sperm-specific PLCZ1 has been identified as the predominant initiator of sperm–oocyte contact. This isoform triggers oocyte activation by releasing IP_3_ and Ca^2+^ during fertilisation [45]. In addition, phospholipase also plays an important role in spermatogenesis. Phospholipase is localised mainly in the acrosomal and post-acrosomal regions. During sperm capacitation and the acrosome reaction, the cellular distribution of phospholipase is altered, which suggests that this enzyme affects sperm quality. According to Wang et al. [46], PLCZ1 plays a key role in maintaining sperm motility in the reproductive system of mice. In turn, Unnikrishnan et al. [47] demonstrated that during capacitation, PLCZ1 is activated through phosphorylation on sperm tyrosyl residues and the interactions with Na/K ATPase α4 (ATP1A) and the epidermal growth factor receptor (EGFR). Therefore, abnormalities in PLCZ1 conformation can lead to infertility. In the present study, the discussed enzyme had a molecular weight of 73.5 kDa (MASCOT), but the weight calculated in the Multi-Analyst program was much lower, probably because PLCZ1 was broken down into smaller subunits during semen processing or interactions with reagents. The phosphorylation of PLCZ1 was more pronounced in white semen, which could indicate that this enzyme plays an essential role in the acquisition of potential fertilising capacity by spermatozoa. In this study, various actin isoforms, actin-binding proteins, and proteins involved in actin polymerisation and depolymerisation, including Rho GTPases, cap proteins, gelsolin, and myosin, were identified using spectrometric methods. Actin is a cytoskeletal protein directly involved in the acquisition of progressive motility, hyperactivated motility, and acrosomal exocytosis in mammalian spermatozoa. There is also evidence to suggest that actin-binding proteins, particularly motor proteins, play an essential role in spermatogenesis [48]. Actin polymerisation/depolymerisation occurs during sperm capacitation, and F-actin depolarisation is crucial for the hyperactivation of male germ cells [49]. Rho proteins CDC42 and RhoA regulate actin polymerisation. Inhibition of CDC42 and RhoA expression disrupts the kinetics of actin polymerisation, capacitation, and the acrosome reaction in various ways. The initiation of actin polymerisation and RhoA activation requires CDC42 activation, and RhoA affects actin polymerisation when CDC42 reaches its maximum expression [50]. The presence of phosphorylated GTPases, actin, and CDC42 suggests that these proteins are involved in cytoskeleton formation and the activation of sperm motility in turkey semen. In boar spermatozoa, fourteen differently expressed and phosphorylated proteins, mainly ODF1, SMCP, AKAP4, FSIP2, and SUN5, were found to play a key role in flagellum development and sperm motility. In turn, the phosphorylation of sperm-specific proteins, including CABYR, ROPN1, CALM1, PRKAR2A, and PRKAR1A, regulates boar sperm motility, mainly via the cAMP/PKA signalling pathway, suggesting that protein phosphorylation may be an important mechanism underlying the development and differentiation of gamete motility [51]. The presence of phosphorylated forms of both ODF and CALM suggests that the mechanisms responsible for the motility of turkey spermatozoa are highly complex.

### 3.4. Effect of Ejaculate Type on the Phosphorylation Profiles of Sperm Proteins

The expression of voltage-dependent anion-selective channel protein 2 (VDAC2), polyubiquitin B (UBB), glyceraldehyde-3-phosphate dehydrogenase (GAPDH), tubulin (TBB), and creatine kinase S-type (CKMT2) was higher in spermatozoa from white semen. VDAC family proteins are involved in many physiological and pathophysiological processes (including energy metabolism and cell apoptosis) by regulating membrane permeability to small ions and molecules (such as Na^+^, Ca^2+^, Cl^−^, ATP, and glutamate). According to recent research, VDACs may play an essential role in spermatogenesis, motility, capacitation, and the acrosome reaction. Additionally, VDAC2 plays a crucial role in apoptosis, gametogenesis, and steroidogenesis pathways, and it serves as a protective mechanism against oxidative stress. The high abundance of VDAC2 and VDAC3 in bull spermatozoa and their ability to gate and bind ATP suggests that these proteins may be involved in directing ATP to the most distally located dynein ATPases in the flagellum and in protecting ATP against premature hydrolysis [52]. Three isoforms of voltage-dependent anion channels have been identified in various regions of the male reproductive tract: VDAC1 is present in Sertoli cells, VDAC2 is located in different parts of spermatozoa (particularly in the outer mitochondrial membrane and outer dense fibres in the axial portion of the flagellum), and VDAC3 is found in the acrosomal region of spermatozoa. HSP70 activates the flagellum by binding to its surface through an interaction with VDAC2. This binding mechanism enables sperm transport to the oviduct [53]. In contrast, high expression of VDAC2 mRNA was found to be positively correlated with low sperm motility. Moreover, abnormal expression of VDAC family proteins may be associated with male infertility caused by idiopathic asthenozoospermia [54]. Han et al. [55] reported that the polyubiquitin gene Ubb is required for fertility in both male and female mice. In the Ubb-null male mice, the arrest of spermatogenesis at the pachytene stage led to the emergence of an azoospermia phenotype. The cited authors also found that 24 testis development-related proteins, including Hsp90aa1, Eef1a1, and Pabpc1, were directly influenced by ubiquitin depletion. Their analyses ultimately demonstrated that Ubb expression is crucial for maintaining testicular RNA-binding proteins and piRNA-metabolic proteins essential for spermatogenesis in mice. Therefore, increased phosphorylation of this protein in turkeys may indicate the formation of morphologically and functionally normal gametes capable of fertilising the oocytes. Mammalian sperm cells utilise carbohydrates as an energy source for the production of ATP through both glycolysis and oxidative phosphorylation. In most animal species, the energy required for sperm motility is generated by the GAPDH isoenzyme, which is also found in somatic cells. D-glyceraldehyde-3-phosphate dehydrogenase is a key glycolytic enzyme that facilitates the oxidative phosphorylation of glyceraldehyde-3-phosphate, producing 1,3-diphosphoglycerate. This intermediate is subsequently utilised by phosphoglycerate kinase to generate ATP [56]. Thus, this enzyme is required for sperm motility and male fertility. GAPDH participates in many processes, including reactions catalysed by protein phosphotransferase and kinase [57]. Tisdale [58] documented that GAPDH is phosphorylated by protein kinase C, specifically iota/lambda, through direct interaction with its regulatory domain. The interaction between beta-tubulin and the membranes depends on phospho-GAPDH and is inhibited by agents that either disrupt Rab2-dependent recruitment of GAPDH to the membrane or interfere with PKC iota/lambda kinase activity. Low expression of major cytoskeletal components in sperm flagella, such as tektins (TEK1, TEK4, and TEK5), outer dense fibre proteins (ODF2), or tubulins (TUBB2C, TUBB2B, and TUBA3C), is associated with reduced sperm motility [10]. In sperm, α- and β-tubulins undergo post-translational modifications, including acetylation, palmitoylation, phosphorylation of tyrosine residues, and polyglutamylation [59]. These changes stabilise microtubules and the associated proteins, affecting flagellar movement [60]. Słowińska et al. [61] identified tau tubulin kinase-2, which regulates tubulin phosphorylation in turkey testes. Both ODF and tubulin proteins were detected in the group of phosphoproteins identified in turkey sperm. Most likely, the degree of their phosphorylation was associated with the achievement of sperm motility. Creatine kinase (CK) activity in human spermatozoa is an indicator of gamete maturity and fertilisation potential. The CK-Mi isoenzyme in human spermatozoa is mitochondria-specific and co-expressed with cytosolic brain-type subunits in energy-demanding tissues, such as the brain, placenta, kidney, testes, sperm, and endothelial cells. MtCK is a central regulator of cellular energy homeostasis and a major target of oxidative stress-induced damage [62,63]. CK-Mi uses mitochondrial ATP to synthesise phosphocreatine, which is transported to the cytoplasm for ATP regeneration by cytosolic isoenzymes. Its activity increases with phosphorylation and decreases with dephosphorylation [64]. The stronger phosphorylation of VDAC2 and CKMT2 in white sperm may be related to more efficient energy management in sperm.

In contrast, the phosphorylation of CKB, TTR, PRDX1, RBP4, and TPI1 was more intense in spermatozoa from yellow semen. The phosphorylation of creatine kinase B (CKB) by protein kinase C enhances its activity by increasing its ability to transfer a high-energy phosphate residue from phosphocreatine to ADP, thereby producing ATP [65]. In turn, AMP-activated protein kinase can regulate CK activity by phosphorylating its serine residues. Both enzymes work closely together under energy stress to compensate for the loss of adenosine triphosphate (ATP) [66]. Research has shown that the activity of both types of CK (CKM and CKB) and their interactions can be biochemical markers of sperm maturity and fertilisation potential. High CK activity in spermatozoa indicates the presence of cytoplasmic residues in their structure and poor gamete quality [67]. Rao et al. [68] suggested a correlation between aberrant removal of cytoplasmic residues and oxidative stress phenomena in spermatozoa. Peroxidative damage is often caused by morphological abnormalities in the midpiece, where excess residual cytoplasm is usually located. This excess can stimulate the production of O^2.−^ by enhancing NADPH availability through increased G6PDH activity. A series of reactions involving the retention of excess residual cytoplasmic enzymes such as CK and G6PDH, increased production of ROS, induction of lipid peroxidation, and sperm dysfunction has been reported [69]. Transthyretin (TTR) is a plasma protein that can bind thyroid hormones (THs) and retinol-binding protein (RBP). It also exhibits proteolytic activity on various substrates, including apolipoprotein A-I (apoA-I) [70]. TTR is a highly conserved 55 kDa homotetrameric protein that undergoes phosphorylation during its gradual conversion into a free form, resulting in a 80 Da change in molecular weight. The oligomeric forms of TTR play a significant role in inducing oxidative stress and may be implicated in various pathophysiological conditions [71]. Moreover, elevated serum levels of TTR during pregnancy interfere with normal reproductive processes [72]. Peroxiredoxins (PRDXs) play a crucial role in antioxidant defence and regulate ROS levels in sperm. Phosphorylation of PrxI on Tyr194 prevents excessive degradation of H_2_O_2_ signalling molecules. This modification is restricted to membrane-associated PrxI, ensuring selective regulation, while most PrxI remains active in H_2_O_2_ elimination [73]. The increased phosphorylation of PrxI may be related to the overproduction of hydrogen peroxide during oxidative processes in yellow semen, while protecting sperm membranes from degradation. Excess hydrogen peroxide is not utilised as a signalling molecule, leading to its accumulation. The presence of peroxiredoxin 1 and 6 in white turkey semen points to effective defence against oxidative stress. A species-specific sperm structure and small ejaculate volume contribute to the complex antioxidant system of turkey semen. Retinol-binding protein 4 (RBP4) is not essential for spermatogenesis; however, it plays a vital role in protecting the testes from the effects of dietary vitamin A deficiency. This protein also plays a key role in Sertoli cells by regulating the synthesis and secretion of testosterone and dihydrotestosterone, and it also transports adipokines and fatty acids [74]. RBP4 is primarily produced in the liver and is secreted by hepatocytes after binding to retinol, forming a complex with TTR. Transthyretin and RBP4 form a higher-molecular-weight complex that prevents renal filtration. Consequently, RBP4 may be present in yellow turkey semen as it binds to circulating TTR or to the metabolic by-products of fatty acids. Metabolomic studies indicate that the processes of lipid synthesis and degradation are altered in yellow turkey semen. The concentrations of intermediary metabolites involved in fatty acid and steroid biosynthesis, such as myristoyl-CoA and farnesyl pyrophosphate (FPP), are elevated in yellow seminal plasma. High levels of myristoyl-CoA can exacerbate lipid peroxidation and reduce semen quality [8]. Cope et al. [75] found that all retinol-binding protein forms undergo in vitro phosphorylation, influencing retinoid action. In contrast, Ninomiya et al. [76] reported no such modifications. Triosephosphate isomerase (TPI) is located in the sperm head and plays a role in sperm binding to the zona pellucida [77]. Its non-enzymatic and “moonlighting” functions have been described in virulence, immunity, spermatozoa differentiation, cell cycle signalling, and neuronal function [78]. In pigs, TPI is highly expressed in the sperm of animals with small litters [79]. According to Vilagran et al. [77], normalised levels of TPI in sperm were significantly higher in porcine ejaculates of low quality. Moreover, TPI was negatively correlated with PMI, sperm morphology, and several motility parameters. Abundant phosphorylation on a single subunit of the TPI dimer has been repeatedly demonstrated. However, the numerous functions ascribed to TPI have not been fully elucidated. Schachner et al. [78] found that asymmetric phosphorylation at Ser20, i.e., on only one subunit, can induce structural changes that promote substrate diffusion into the TPI active site, thereby significantly enhancing its activity. Therefore, increased phosphorylation of TPI in yellow turkey semen may influence its activity and impact the efficiency of the glycolytic pathway in spermatozoa.

## 4. Conclusions

The spermatozoa of turkeys with YSS had lower MDA levels, decreased PMI and MMP, increased antioxidant enzyme activity, and an elevated GSH content. Yellow semen also had a lower percentage of viable sperm cells and a higher percentage of apoptotic and necrotic cells. YSS affected the phosphorylation of serine, threonine, and tyrosine residues in the sperm phosphoproteome. The phosphoproteins identified in turkey sperm are crucial for sperm maturation, motility development, cellular metabolism, protection against oxidative stress, and successful fertilisation. These phosphoproteins are encoded by the following genes: UBB, RPS27A, SPATA18, CDC42, ACTN, TBB, ACTA, ODF2, GAPDH, PGAM, PGK, TPI, ALDOC, LDH, SDHA, FASN, APOA, CKM, GOT, CA, CALM, TRFC, FTH, HSPA, HSP, GST, PRDX, QSOX, ASTL, ANXA, ACR, GSN, VDAC, and PLCZ1. This study demonstrated that phosphorylation patterns in proteins such as VDAC2, CKMT2, CKB, TTR, PRDX, RBP4, and TPI may influence sperm motility and fertility. The proteins encoded by TBB, CKMT2, VDAC2, UBB, and GAPDH were more intensely phosphorylated in white semen, whereas those encoded by CKB, TTR, PRDX1, RBP4, and TPI1 were more intensely phosphorylated in yellow semen. The increased phosphorylation of VDAC2, CKMT2, and GAPDH in white semen points to more efficient energy management, while altered phosphorylation (particularly of PRDX, CKB, TTR, and TPI) in yellow semen may affect antioxidant and lipid metabolism and energy acquisition.

## 5. Materials and Methods

### 5.1. Materials

The experimental material consisted of 100 ejaculates from BIG-6 (Aviagen, Huntsville, AL, USA) male turkeys (*Meleagris gallopavo*). The ejaculates were collected from birds aged 39 to 42 weeks. The turkeys originated from a parent flock on the GERCZAK farm (Nord-Pol Hatchery, Iława, Poland) in Kozia Góra, Warmian-Masurian Voivodeship, Poland. The birds were kept under the highest zoohygienic and biosecurity standards. Semen was collected from each bird using the Burrows and Quinn method [80], and each sample was placed in a separate syringe. Prior to dilution, ejaculates were classified into two quality groups based on colour. Ejaculates with a white or pearly colour were classified as white (W = 50), while those with a yellow or yellowish colour were classified as yellow (YSS; Y = 50). A qualified farm employee assessed semen colour against a blue background, assigning each ejaculate to the appropriate group. The quality of each ejaculate was then verified in the laboratory by measuring the total protein content of seminal plasma using the Bradford method. Semen containing up to 10 mg/mL of total protein in the seminal plasma was classified as white, while semen containing more than 10 mg/mL of total protein was classified as yellow. Total protein content was determined by the method proposed by Thurston et al. [2] and adjusted for the Bradford method, which often yields lower results than that of Thuston et al. In the next step, semen was diluted with a commercial extender (Extendyl, IMV Technologies, L’Aigle, France) (*v*/*v*, 1:1), and the samples were placed in a thermobox with a temperature of 38–39 °C and transported to the Department of Animal Biochemistry and Biotechnology at the University of Warmia and Mazury in Olsztyn for further analysis.

Sperm concentration, sperm motility parameters, viability and functionality of sperm were assessed, and the samples were centrifuged at 10,000× *g* for 10 min. The obtained seminal plasma was removed, and the precipitated spermatozoa were washed twice with a 0.85% NaCl solution and centrifuged again under identical conditions. The pellet was resuspended in TBS with 1% SDS (50 mM TRIS, 150 mM NaCl, 1% SDS; pH 7.5) to a volume of 1.5 mL. The Protease and Phosphatase Inhibitor Cocktail (Sigma-Aldrich, Merck, Burlington, MA, USA) was added to the sperm extracts (*v*/*v*, 1:100). The samples were stored at −80 °C until further analysis.

### 5.2. Determination of Sperm Concentration

Sperm concentration was determined using a light microscope (Olympus CH30, Olympus, Tokyo, Japan; 40× magnification) and a Bürker counting chamber. The results were used to calculate the average number of spermatozoa per one large square in the counting chamber. In the following step, all samples were diluted to a concentration of 200 × 10^6^ spermatozoa/mL with Extendyl (IMV Technologies, L’Aigle, France).

### 5.3. Determination of Sperm Motility Parameters (CASA)

Sperm motility was assessed with the Computer-Aided Sperm Analysis (CASA) system (Hamilton-Thorne Research, IVOS version 12.3; Beverley, MA, USA). The CASA settings were configured according to the Hamilton Thorne technical guide (v. 12.3) for turkey spermatozoa, as follows: 60 frames were acquired at a frequency rate of 60 Hz, with a minimum cell contrast of 35 and a minimum cell size of 5 pixels. The VAP threshold was set at 50 μm/s, the STR threshold at 80.0%, the VAP cutoff at 30.0 μm/s, and the VSL cutoff at 15.0 μm/s. To estimate sperm motility, the sample was diluted to a concentration of 30 × 10^6^ spermatozoa/mL with an activation buffer (50 mM TRIS, 130 mM NaCl, 2 mM CaCl_2_, 10 mM glucose, 0.5% BSA; pH 7.4). The prepared solutions were placed in a thermostat (Thermo Block TDB-120, Biosan, Riga, Latvia) at 38 °C for 15 min. Subsequently, 10 µL of the resulting mixture was placed in a Makler chamber to assess the following sperm motility and kinetic parameters: percentage of motile spermatozoa (TMOT), percentage of progressively motile spermatozoa (PMOT), average path velocity (VAP), straight-line velocity (VSL), curvilinear velocity (VCL), amplitude of lateral head displacement (ALH), beat cross frequency (BCF), straightness (STR), and linearity (LIN).

### 5.4. Assessment of Plasma Membrane Integrity

Sperm plasma membrane integrity (PMI) was assessed by double staining with SYBR-14 fluorochromes and propidium iodide (PI) (Live/Dead Sperm Viability Kit, Thermo Fisher Scientific, Waltham, MA, USA) according to the method proposed by Garner and Johnson [81] with some modifications [82]. Semen samples were brought to a concentration of 30 × 10^6^ sperm/mL using the HEPES/BSA buffer (10 mM HEPES, 130 mM NaCl, 4 mM KCl, 14 mM fructose, 1 mM CaCl_2_, 0.5 mM MgCl_2_, and 0.1% BSA; pH 7.4). Then, 1 µL of SYBR-14 solution (1 mM SYBR-14 in DMSO) was added to 100 µL of the test sample and incubated in a thermostat (Thermo Block TDB-120, Biosan, Riga, Latvia) for 10 min at 38 °C. Subsequently, 1 µL of propidium iodide solution (2.4 µM PI in Tyrode’s solution) was added to the samples and incubated under the same conditions for 10 min. The solutions were assessed under a fluorescence microscope (Olympus CH 30 RF-200, Olympus, Tokyo, Japan). A 10 µL sample was placed on a slide and covered with a coverslip. At least 100 spermatozoa were assessed in ten randomly selected fields of view. Spermatozoa with green heads (SYBR-14) were considered to have intact membranes in the head region, whereas spermatozoa with red heads (PI) were considered to have damaged membranes. The results were presented as the percentage of spermatozoa (%) with intact membranes in the acrosomal region.

### 5.5. Assessment of Mitochondrial Function in Spermatozoa

Sperm mitochondrial membrane potential (MMP) was assessed using a commercial reagent kit containing 5,5′,6,6′-tetrachloro-1,1′,3,3′-tetraethylbenzimidazolyl-carbocyanine iodide (JC-1) and propidium iodide (PI) (Mitochondrial Membrane Potential Probe, Thermo Fisher Scientific, Waltham, MA, USA) according to the method described by Thomas et al. [83]. Semen samples were diluted to a concentration of 30 × 10^6^ sperm/mL using the HEPES/BSA buffer (10 mM HEPES, 130 mM NaCl, 4 mM KCl, 14 mM fructose, 1 mM CaCl_2_, 0.5 mM MgCl_2_, and 0.1% BSA; pH 7.4). Samples of 100 µL each were combined with 1 µL of JC-1 stock dye solution and incubated in a thermostat for 10 min at 38 °C (Thermo Block TDB-120, Biosan, Riga, Latvia). Then, 1 µL PI was added to the semen solution and incubated under the same conditions. The prepared samples were assessed under a fluorescence microscope (Olympus CH 30 RF-200, Olympus, Tokyo, Japan) using a green DMG filter. A 10 µL sample was placed on a slide and covered with a coverslip. At least 100 spermatozoa in ten randomly selected fields of view were assessed. Spermatozoa showing orange–red fluorescence (JC-1) in the midpiece were considered viable with high MMP, while spermatozoa showing red fluorescence (PI) in the head and green fluorescence in the midpiece were classified as dead. The results were presented as the percentage of spermatozoa (%) with functional mitochondria.

### 5.6. Assessment of Pro-Apoptotic Changes in Spermatozoa

Apoptotic changes in spermatozoa were assessed using a commercial mixture of fluorochrome dyes: YO-PRO-1 and propidium iodide (PI) (Vybrant Apoptosis Assay Kit #4, Thermo Fisher Scientific, Waltham, MA, USA) [84,85]. Semen samples were brought to a concentration of 30 × 10^6^ sperm/mL using the HEPES/BSA buffer (10 mM HEPES, 130 mM NaCl, 4 mM KCl, 14 mM fructose, 1 mM CaCl_2_, 0.5 mM MgCl_2_, and 0.1% BSA; pH 7.4). Subsequently, 0.5 µL of YO-PRO-1 dye solution and 0.5 µL of PI solution were added and incubated at 38 °C for 10 min. The prepared samples were assessed under a fluorescence microscope (Olympus CH 30 RF-200, Olympus, Tokyo, Japan). A 10 µL sample was placed on a slide and covered with a coverslip. At least 100 spermatozoa were assessed in ten randomly selected fields of view. Spermatozoa with red-stained heads were classified as necrotic, whereas those with red–green heads were regarded as early necrotic spermatozoa. In contrast, spermatozoa with green heads were classified as early apoptotic. Spermatozoa with uncoloured heads were classified as viable without apoptotic changes. The results were presented as the percentage of viable, apoptotic, and necrotic spermatozoa (%).

### 5.7. Estimation of the Percentage of Sperm Producing Nitric Oxide

The percentage of sperm cells producing nitric oxide (NO) was determined with the use of diacetate-4,5-diaminofluorescein fluorescent dye (DAF-2DA) (Sigma-Aldrich, Merck, Burlington, MA, USA) according to the methodology proposed by Lampiao et al. [86] with some modifications. Semen samples were diluted to a concentration of 30 × 10^6^ with the HEPES/BSA buffer (10 mM HEPES, 130 mM NaCl, 4 mM KCl, 14 mM fructose, 1 mM CaCl_2_, 0.5 mM MgCl_2_, and 0.1% BSA; pH 7.4). Then, 100 µL of DAF-2DA solution (20 µM DAF-2DA in PBS) was added to 100 µL of the sample and incubated at 38 °C for two hours in the dark. The prepared samples (10 µL each) were placed on slides, covered with a coverslip, and analysed under a fluorescence microscope (Olympus CH 30 RF-200, Olympus, Tokyo, Japan) with a blue DMB filter. At least 100 spermatozoa were evaluated in ten randomly selected fields of view. Spermatozoa exhibiting blue–green fluorescence in any segment were considered to be NO-producing cells. The result was expressed as the percentage (%) of NO-producing spermatozoa in one field of view.

### 5.8. Antioxidant Capacity of Sperm

The activity of antioxidant enzymes, GSH content, and MDA levels were evaluated in sperm extracts obtained from each ejaculate. Samples were analysed in duplicate. The activity of specific enzymes was adjusted to the sperm concentration and expressed as U/10^9^ sperm for SOD and GPx, and µM/min/10^9^ sperm for CAT; the GSH content and MDA levels were also adjusted to the sperm concentration and expressed in µM/10^9^ sperm.

#### 5.8.1. Determination of Superoxide Dismutase (SOD) Activity

Superoxide dismutase (SOD) activity was determined using a commercial Ransod kit (Randox, Crumlin, UK) according to the manufacturer’s instructions. The volume of each test sample was 50 µL. In this assay, xanthine and xanthine oxidase generated superoxide anion radicals, which subsequently reacted with 2-(4-iodophenyl)-3-(4-nitrophenol)-5-phenyltetrazolium chloride (INT) to produce red formazan. The intensity of the resulting colour corresponded to SOD activity and was measured spectrophotometrically at 505 nm. One unit of SOD was defined as the amount required to inhibit INT reduction by 50% per minute at 37 °C.

#### 5.8.2. Determination of Glutathione Peroxidase (GPx) Activity

Glutathione peroxidase (GPx) activity was determined using a Ransel kit (Randox, Crumlin, UK) according to the manufacturer’s recommendations. The volume of each test sample was 20 µL. The assay relied on GPx’s ability to catalyse the oxidation of GSH by cumene hydroperoxide. Simultaneously, glutathione disulphide (GSSG) was reduced back to GSH, while NADPH was oxidised to NADP+. The subsequent decrease in absorbance was recorded at 340 nm and 37 °C (pH 7.2).

#### 5.8.3. Determination of Catalase Activity (CAT)

Catalase (CAT) activity was determined using a commercial Catalase Assay Kit reagent kit (Sigma-Aldrich, Merck, Burlington, MA, USA) according to the manufacturer’s instructions. The volume of each test sample was 60 µL. Catalase rapidly decomposed H_2_O_2_ as its substrate. The remaining H_2_O_2_ reacted with quinoneimine dye, producing a coupling product that was measured spectrophotometrically at 520 nm. One unit of CAT was defined as the quantity that decomposes 1 M of H_2_O_2_ per minute at a substrate concentration of 50 mM H_2_O_2_ at 25 °C (pH 7.0).

#### 5.8.4. Determination of Reduced Glutathione (GSH) Content

Reduced glutathione (GSH) content was determined using the Bioxytech GSH-400 reagent kit (AOXRE Bio-Sciences, Burlingame, CA, USA) according to the manufacturer’s recommendations. First, the samples had to be deproteinised by adding 0.3 mL of 5% metaphosphoric acid to 0.1 mL of each sperm extract. After incubation for 30 min, the samples were centrifuged at 10,000× *g* for 10 min at room temperature. The resulting supernatant with a volume of 50 µL was used for further analysis. In the initial step, substitution products (thioethers) were formed through the reaction between a patented reagent, R1 (4-chloro-1-methyl-7-trifluoromethyl-quinolinium methylsulfate), and mercaptans (RSH) present in the sample. The thioethers were then converted into chromophoric thione in a reaction facilitated by reagent R2 (30% NaOH). The resulting product exhibited maximum absorbance at 400 nm and 25 °C.

#### 5.8.5. Determination of Malondialdehyde (MDA) Levels

Malondialdehyde (MDA) levels were determined using the Bioxytech MDA-586 kit (AOXRE Bio-Sciences, Burlingame, CA, USA) according to the manufacturer’s protocol. Samples for MDA analysis were prepared by adding 1 mL of 0.85% NaCl to sperm suspensions (5 × 10^7^ sperm cells). After two rounds of centrifugation at 3000× *g* for 10 min at 4 °C, the samples were homogenised by two freeze–thaw cycles. Prior to MDA determination, the samples were subjected to centrifugation at 10,000× *g* for 10 min, and the resulting supernatant with a volume of 200 µL was used for further analysis. The assay relied on the reaction between N-methyl-2-phenylindole (R1, NMPI) and MDA at 45 °C. During this process, one MDA molecule interacted with two NMPI molecules to produce a stable carbocyanine dye. The concentration of MDA in the sample was determined by measuring the absorbance of the probe and comparing it to a standard curve at 586 nm in the MDA-586 assay.

### 5.9. Evaluation of Total Protein Content in Sperm Extracts

Total protein content was measured using the Bradford reagent (Sigma-Aldrich, Merck, Burlington, MA, USA), following the manufacturer’s specified procedure.

### 5.10. Isolation of Phosphoproteins by Immobilised Fe^3+^ Affinity Chromatography on PHOS-Select Iron Affinity Gel Beads

Phosphoproteins were isolated by immobilised Fe^3+^ affinity chromatography using PHOS-Select Iron Affinity Gel beads (Sigma-Aldrich, Merck, Burlington, MA, USA). We placed 40 µL of the gel beads in a test tube and equilibrated that three times with 500 µL of buffer A (250 mM acetic acid, 30% acetonitrile; pH 2.9). After the addition of each reagent, the samples were stirred and centrifuged in a Microfuge 22R microcentrifuge (Beckman Coulter, Brea, CA, USA) at 10,000× *g* for 30 s. Each time, the obtained supernatant was removed. A 500 µL sample standardised to a protein content of 2 mg/mL was added to the enucleated bead with buffer A. The tubes were then placed on a laboratory cradle, and the contents were stirred at room temperature for 30 min. The tubes were centrifuged at 10,000× *g* for 30 s. The resulting supernatant was removed, and 500 µL of buffer A was added. The samples were centrifuged again under identical conditions, and the obtained supernatants were decanted. Subsequently, 500 µL of deionised water was added to the pellets, and the tubes were centrifuged. Once again, the supernatant was removed, and the isolated phosphoproteins were eluted. For this purpose, 100 µL of buffer B (400 mM ammonia) was added to the precipitate. The samples were centrifuged again under identical conditions. The resulting supernatants were transferred to fresh tubes and frozen at −80 °C.

### 5.11. Determination of the Molecular Weight of Sperm Phosphoproteins by One-Dimensional SDS Gel Electrophoresis (SDS-PAGE)

Sperm phosphoproteins were separated on 12% polyacrylamide gels [87]. The samples were prepared by adding 10 µL of the solution of the isolated phosphoproteins to 2 µL of a loading buffer (1 M Tris-HCl. 20% SDS, 20% glycerol, 2% β-mercaptoethanol, 2% bromophenol blue; pH 6.8). The prepared samples were placed in individual wells in the gel. Proteins were separated in a Mini-Protean II system (Bio-Rad Laboratories, Hercules, CA, USA) by filling the chamber with the SDS-PAGE electrophoresis buffer (50 mM Tris, 250 mM glycine, 0.5% SDS; pH 8.3). The voltage in the electrophoresis system was initially set to 80 V and increased to 130 V after the samples had migrated through the gel. The electrophoretic mobility of the separated phosphoproteins was determined using Precision Plus Protein Standards (Bio-Rad Laboratories, Hercules, CA, USA). After separation, the gels were stained with Coomassie Brilliant Blue R-250 solution (30% methanol, 5% acetic acid, 0.1% CBB R-250) (Sigma-Aldrich, Merck, Burlington, MA, USA) for 24 h. The gels were destained with 5% methanol and 7% acetic acid. Destained gels were subjected to a densitometric analysis using Multi-Analyst version 1.1. software (Bio-Rad Laboratories, Hercules, CA, USA).

### 5.12. Electrotransfer and Immunodetection of Sperm Phosphoproteins

Phosphoproteins separated by denaturing gel electrophoresis (SDS-PAGE) were electrotransferred to membranes using 0.5 µL of Biotinylated Molecular Weight Marker standards (Sigma-Aldrich, Merck, Burlington, MA, USA). Polyvinylidene fluoride (PVDF) membranes (Merck Millipore, Burlington, MA, USA) were used for this purpose. The membranes were immersed in methanol and placed in a transfer buffer (50 mM Tris, 250 mM glycine, 20% methanol). Filter paper was trimmed to the size of the gel and applied to the gel for 15 min. The samples were subjected to semi-dry electroblotting in a Semi-Dry Blotter (Sigma-Aldrich, Merck, Burlington, MA, USA) at a constant current of 1 mA/cm^2^ of membrane area for one hour. The membranes were then incubated at room temperature for two hours in a wash solution (20 mM Tris, 136 mM NaCl, 0.1% Tween-20; pH 7.5) containing 2% bovine serum albumin (BSA). After incubation, PVDF membranes were washed three times (5 min each) with a wash buffer. They were incubated for at least 12 h at 4 °C on a laboratory cradle in a wash buffer (50 mL) containing selected biotinylated monoclonal antibodies. Three types of antibodies were used: anti-phosphoserine (diluted 1:300,000), anti-phosphothreonine (diluted 1:60,000), and anti-phosphotyrosine (diluted 1:50,000). On the following day, the membranes were washed with a wash buffer and incubated for 1 h at room temperature in a buffer containing 50 µL of streptavidin-alkaline phosphatase conjugate (Vector Laboratories, Newark, CA, USA). After incubation, the membranes were washed with a wash buffer (3 × 5 min) and deionised water (1 min). The membranes were stained by adding 200 µL of a mixture of nitro blue tetrazolium (NBT) and 5-bromo-4-chloro-3-indolyl-phosphate (BCIP) (NBT/BCIP Stock Solution, Sigma-Aldrich, Merck, Burlington, MA, USA) to 10 mL of an alkaline phosphatase buffer (100 mM Tris, 100 mM NaCl; pH 9.5). The membranes were left in the mixture until the stained segments achieved the optimal colour and were then dried in open air. The molecular weights of the stained phosphoprotein fractions were determined by analysing the membranes using Multi-Analyst version 1.1. software (Bio-Rad Laboratories, Hercules, CA, USA).

### 5.13. Identification of Sperm Phosphoproteins by Nano LC-MS/MS

Excised sections of SDS-PAGE gels were destained, reduced, alkylated, and digested. Initially, gel pieces were immersed in 50 µL of ammonium carbonate solution (100 mM) and shaken in a thermoblock for 10 min. Then, 50 µL of acetonitrile was added, and the tubes were shaken at 600 rpm for 10 min. The supernatant was removed, and gel fragments were dehydrated by adding 100% acetonitrile and shaking again under the same conditions. In the next step, gel fragments were immersed in 50 µL of aqueous dithiothreitol (DTT) solution. Reduction was carried out for more than 10 min at a temperature of 90 °C. Then, 50 µL of 5 mM iodoacetamide solution in 100 mM carbon buffer was added. The gels were alkylated in a thermoblock at 90 °C and 600 rpm for 10 min. At the end of the alkylation process, the iodoacetamide solution was removed, and gel pieces were dehydrated with acetonitrile. In the next step, 1.5 pmol of trypsin (Promega, Madison, WI, USA) was added, and the samples were rehydrated for 5 min. The samples were incubated overnight in carbonate buffer in a thermoblock set to 37 °C. On the following day, peptides were extracted once with 50 mM carbonate buffer and twice with 5% formic acid solution in 50% acetonitrile. Each extraction step lasted 15 min and was carried out at 37 °C. The fractions containing eluted peptides were combined. The extract was freeze-dried and dissolved in 30 µL of a solution of 4% acetonitrile with 0.1% formic acid. The nano LC-MS/MS analysis was conducted in an Ultimate 3000 HPLC/UPLC system (Thermo Fisher Scientific, Waltham, MA, USA) connected online to an Exploris 240 mass spectrometer (Thermo Fisher Scientific, Waltham, MA, USA) in positive ion mode. Samples of 5 µL were injected into the LC system composed of an RP C18 precolumn (0.3 × 5 mm, 5 µm particle size) (Thermo Fisher Scientific, Waltham, MA, USA) and an RP C18 column (15 cm, 3 µm particle size, 75 µm diameter) (Thermo Fisher Scientific, Waltham, MA, USA). The mobile-phase flow rate was 300 mL/min. The samples were eluted using a 33 min gradient of solutions A (0.1% fluoroacetic acid in water) and B (0.1% fluoroacetic acid in acetonitrile). The gradient was as follows: t = 0 min, 8% B; 4 min, 8% B; 30 min, 35% B; 30 min 30 s, 90% B; 31 min 3 s, 90% B; 32 min, 8% B, 33 min, 8% B.

### 5.14. Functional Analysis of the Identified Sperm Phosphoproteins

The ontology of the genes (GO) encoding selected phosphoproteins in turkey semen was determined in the first step of the analysis. The ShinyGo programme (version 0.77, http://bioinformatics.sdstate.edu/go/; accessed on 15 January 2025) was used to classify proteins according to their biological functions. In the second step, the interactions between turkey sperm phosphoproteins were analysed in the STRING bioinformatics programme (version 11.5, http://string-db.org; accessed on 15 January 2025). The minimum required interaction score was ≥0.4 (mean confidence level). All interactions were predicted with the use of the *Homo sapiens* database (the largest and continuously updated gene database). Cell signalling is primarily determined by the interactions between the amino acid residues of neighbouring proteins; therefore, protein–protein interactions were also examined.

### 5.15. Statistical Analysis

Statistical analysis was performed using Statistica software (version 13.3., StatSoft, TIBCO Software, Palo Alto, CA, USA). The results were presented as arithmetic means ± standard errors of the mean (SEM). The optical density (OP) of sperm phosphoproteins isolated by immunodetection methods was determined by the Mann–Whitney U test. The remaining parameters were processed by one-way analysis of variance (ANOVA) and Tukey’s test. The presence of correlations between the analysed parameters was determined by calculating Pearson’s correlation coefficients.

## Figures and Tables

**Table 1:** Biological parameters of spermatozoa derived from white and yellow semen.

	White	Yellow
TMOT (%)	68.43 ± 3.58 ^a^	57.43 ± 3.91 ^b^
PMOT (%)	35.22 ± 2.34	32.37 ± 3.37
VAP (µm/s)	77.68 ± 1.16	80.19 ± 1.95
VSL (µm/s)	66.55 ± 1.45 ^b^	72.44 ± 2.14 ^a^
VCL (µm/s)	113.07 ± 1.59 ^A^	105.23 ± 2.17 ^B^
ALH (µm)	3.63 ± 0.07	3.59 ± 0.11
BCF (Hz)	17.64 ± 0.67 ^A^	14.25 ± 0.83 ^B^
STR (%)	84.60 ± 0.87 ^B^	89.37 ± 0.90 ^A^
LIN (%)	62.29 ± 1.43 ^B^	70.69 ± 1.69 ^A^
Sperm concentration (×10^9^)	3.51 ± 0.12 ^A^	2.18 ± 0.13 ^B^
PMI (%)	85.78 ± 0.82 ^a^	81.58 ± 1.56 ^b^
MMP (%)	83.73 ± 1.02 ^A^	79.01 ± 1.44 ^B^
NO-producing sperm (%)	42.65 ± 3.36	44.09 ± 3.75
Viable sperm (%)	85.91 ± 1.01 ^A^	75.66 ± 0.71 ^B^
Pro-apoptotic spermatozoa (%)	12.16 ± 0.95 ^B^	21.03 ±0.63 ^A^
Necrotic spermatozoa (%)	1.93 ± 0.33 ^B^	3.32 ± 0.27 ^A^
SOD activity (U/10^9^ sperm.)	0.42 ± 0.04 ^B^	0.86 ± 0.14 ^A^
GPx activity (U/10^9^ sperm.)	99.36 ± 13.39 ^b^	168.68 ± 30.11 ^a^
CAT activity (µM/min/10^9^ sperm.)	1.84 ± 0.78	5.95 ± 2.49
GSH content (µM/10^9^ sperm.)	85.39 ± 6.39 ^B^	136.13 ± 21.69 ^A^
MDA levels (µM/10^9^ sperm.)	15.31 ± 1.87	12.71 ± 1.72

^A,B^—highly significant differences (*p* ≤ 0.001). ^a,b^—significant differences (*p* ≤ 0.05). TMOT—total motility, PMOT—progressive motility, VAP—average path velocity, VSL—straight-line velocity, VCL—curvilinear velocity, ALH—amplitude of lateral head displacement, BCF—beat cross frequency, STR—straightness, LIN—linearity coefficient, PMI—plasma membrane integrity, MMP—mitochondrial membrane potential, SOD—superoxide dismutase, GPx—glutathione peroxidase, CAT—catalase, GSH—glutathione, MDA—malondialdehyde.

**Table 2:** Coefficients of correlation between the antioxidant status and motility parameters of spermatozoa derived from white semen.

	TMOT (%)	PMOT (%)	VAP (µm/s)	VSL (µm/s)	VCL (µm/s)	ALH (µm)	BCF (Hz)	STR (%)	LIN (%)
SOD activity (U/10^9^ sperm.)	−0.11	−0.02	0.10	0.12	−0.06	−0.05	0.04	0.11	0.11
GPx activity (U/10^9^ sperm.)	0.02	0.10	−0.26	−0.25	0.05	0.06	0.32	−0.22	−0.24
CAT activity (µM/min/10^9^ sperm.)	0.13	0.29	0.15	0.13	0.14	−0.05	0.22	0.07	0.05
GSH content (µM/10^9^ sperm.)	−0.16	−0.15	−0.31	−0.21	−0.21	−0.19	0.27	−0.03	−0.08
MDA levels (µM/10^9^ sperm.)	−0.10	−0.04	−0.02	−0.03	−0.01	0.11	0.10	−0.04	−0.06

TMOT—total motility, PMOT—progressive motility, VAP—average path velocity, VSL—straight-line velocity, VCL—curvilinear velocity, ALH—amplitude of lateral head displacement, BCF—beat cross frequency, STR—straightness, LIN—linearity coefficient, SOD—superoxide dismutase, GPx—glutathione peroxidase, CAT—catalase, GSH—glutathione, MDA—malondialdehyde.

**Table 3:** Coefficients of correlation between the antioxidant status and motility parameters of spermatozoa derived from yellow semen.

	TMOT (%)	PMOT (%)	VAP (µm/s)	VSL (µm/s)	VCL (µm/s)	ALH (µm)	BCF (Hz)	STR (%)	LIN (%)
SOD activity (U/10^9^ sperm.)	0.15	0.03	−0.07	−0.02	−0.28	−0.05	−0.21	0.18	0.14
GPx activity (U/10^9^ sperm.)	0.12	0.13	−0.33	−0.24	−0.35	0.19	−0.02	0.03	−0.04
CAT activity (µM/min/10^9^ sperm.)	0.52 *	0.56 *	−0.15	−0.13	0.05	−0.17	0.63	−0.03	−0.18
GSH content (µM/10^9^ sperm.)	0.21	0.24	0.62 *	0.63 *	0.14	0.48	0.06	0.51 *	0.52 *
MDA levels (µM/10^9^ sperm.)	0.20	0.32	0.24	0.38	0.51 *	−0.19	−0.28	0.51 *	0.60 *

Values given with asterisks are statistically significant at *p* ≤ 0.05. TMOT—total motility, PMOT—progressive motility, VAP—average path velocity, VSL—straight-line velocity, VCL—curvilinear velocity, ALH—amplitude of lateral head displacement, BCF—beat cross frequency, STR—straightness, LIN—linearity coefficient, SOD—superoxide dismutase, GPx—glutathione peroxidase, CAT—catalase, GSH—glutathione, MDA—malondialdehyde.

**Table 4:** Coefficients of correlation between the antioxidant status and viability parameters of spermatozoa derived from white semen.

	Sperm Concentration (×10^6^ sperm./mL)	PMI (%)	MMP (%)	NO (%)	Viable (%)	Pro-Apoptotic (%)	Necrotic (%)
SOD activity (U/10^9^ sperm.)	−0.44 *	0.15	0.14	0.44 *	0.28	−0.33	0.20
GPx activity (U/10^9^ sperm.)	−0.37 *	0.10	0.08	0.06	0.15	−0.15	0.00
CAT activity (µM/min/10^9^ sperm.)	0.08	0.08	0.28	0.00	−0.43 *	0.45 *	−0.08
GSH content (µM/10^9^ sperm.)	−0.14	0.09	0.02	−0.18	0.09	−0.04	−0.17
MDA levels (µM/10^9^ sperm.)	0.36 *	−0.02	0.14	0.05	0.00	0.01	−0.06

Values given with asterisks are statistically significant at *p* ≤ 0.05. SOD—superoxide dismutase, GPx—glutathione peroxidase, CAT—catalase, GSH—glutathione, MDA—malondialdehyde.

**Table 5:** Coefficients of correlation between the antioxidant status and viability parameters of spermatozoa derived from yellow semen.

	Sperm Concentration (×10^6^ sperm./mL)	PMI (%)	MMP (%)	NO (%)	Viable (%)	Pro-Apoptotic (%)	Necrotic (%)
SOD activity (U/10^9^ sperm.)	−0.25	0.32	0.44	0.33	0.04	−0.20	0.33
GPx activity (U/10^9^ sperm.)	−0.35	0.14	0.10	0.18	−0.17	0.22	−0.12
CAT activity (µM/min/10^9^ sperm.)	−0.25	0.16	0.28	−0.20	0.09	0.04	−0.26
GSH content (µM/10^9^ sperm.)	−0.07	−0.14	−0.10	0.34	−0.03	−0.10	0.26
MDA levels (µM/10^9^ sperm.)	0.38 *	−0.37	−0.34	0.57 *	−0.04	−0.09	0.19

Values given with asterisks are statistically significant at *p* ≤ 0.05. SOD—superoxide dismutase, GPx—glutathione peroxidase, CAT—catalase, GSH—glutathione, MDA—malondialdehyde.

**Table 6:** The average optical density (OD) of turkey sperm proteins from white and yellow semen selected for identification.

Protein Band [kDa]	White	Yellow
52	0.48	0.32
51	0.43	0.23
49	0.24	0.45
43	0.30	0.44
40	0.49	0.27
29	0.38	0.25
24	0.33	0.16
18	0.22	0.29
15	0.24	0.28
13	0.23	0.35

**Table 7:** Optical density (OD) of turkey sperm proteins phosphorylated on serine (SER), threonine (THR), and tyrosine (TYR) residues.

Protein Band	Molecular Weight (Multi-Analyst)	Residue	Type of Semen	Arithmetic Mean	±SEM
15	52 kDa	SER	W	0.17	0.03
			Y	0.16	0.04
		THR	W	0.16 *	0.02
			Y	0.27 *	0.03
		TYR	W	0.14	0.04
			Y	0.20	0.03
16	51 kDa	SER	W	0.18	0.03
			Y	0.17	0.04
		THR	W	0.17	0.03
			Y	0.27	0.03
		TYR	W	0.16	0.05
			Y	0.21	0.03
17	49 kDa	SER	W	0.18	0.03
			Y	0.15	0.03
		THR	W	0.18	0.03
			Y	0.27	0.03
		TYR	W	0.15	0.04
			Y	0.20	0.03
18	43 kDa	SER	W	0.17	0.03
			Y	0.14	0.02
		THR	W	0.16 *	0.02
			Y	0.25 *	0.02
		TYR	W	0.13	0.03
			Y	0.18	0.02
19	40 kDa	SER	W	0.20	0.04
			Y	0.18	0.03
		THR	W	0.17 *	0.03
			Y	0.27 *	0.03
		TYR	W	0.16	0.04
			Y	0.20	0.03
20	29 kDa	SER	W	0.31	0.08
			Y	0.40	0.09
		THR	W	0.30	0.06
			Y	0.42	0.07
		TYR	W	0.29	0.07
			Y	0.33	0.06
21	24 kDa	SER	W	0.11	0.03
			Y	0.09	0.02
		THR	W	0.16	0.03
			Y	0.19	0.01
		TYR	W	0.11	0.02
			Y	0.10	0.02
22	18 kDa	SER	W	0.07	0.01
			Y	0.05	0.01
		THR	W	0.13	0.03
			Y	0.18	0.01
		TYR	W	0.07	0.02
			Y	0.08	0.01
23	15 kDa	SER	W	0.10	0.03
			Y	0.12	0.04
		THR	W	0.15	0.04
			Y	0.18	0.01
		TYR	W	0.08	0.03
			Y	0.10	0.02
24	13 kDa	SER	W	0.04	0.01
			Y	0.05	0.02
		THR	W	0.14	0.03
			Y	0.16	0.01
		TYR	W	0.07	0.02
			Y	0.06	0.01

Values given with asterisks are statistically significant at *p* ≤ 0.05. W—white semen. Y—yellow semen.

## Data Availability

The original contributions presented in this study are included in the article/Supplementary Materials. Further inquiries can be directed to the corresponding author.

## Supplementary Materials

The following supporting information can be downloaded at https://www.mdpi.com/article/10.3390/ijms26083467/s1.
