# Supplementary material for: Does the Type of Semen Affect the Phosphoproteome of Turkey (Meleagris gallopavo) Spermatozoa?
Source: Int J Mol Sci. 2025 Apr 8;26(8):3467. doi: 10.3390/ijms26083467 (PMC12027420; doi:10.3390/ijms26083467)
Supplement: Supplementary file 1 [file ijms-26-03467-s001.zip › ijms-3517189-supplementary.pdf]

Table S1. Phosphoproteins identified in turkey spermatozoa by SDS-PAGE and nano LC-MS/MS.

No	Identified protein	Gene	Species	Molecular weight in Multi-Analyst	Molecular weight (Da)	Number of compatible peptides	Sequence coverage [%]	Score
**1**	Tubulin beta-3 chain	*TBB3*	*Gallus gallus*	52 kDa	50285	116	44	3511
	Tubulin beta-1 chain	*TBB1*	*Gallus gallus*		50377	82	33	2149
	Tubulin beta-2 chain	*TBB2*	*Gallus gallus*		50377	82	20	2149
	Tubulin beta-4 chain	*TBB4*	*Gallus gallus*		50844	81	19	2117
	Tubulin beta-5 chain	*TBB5*	*Gallus gallus*		50395	78	22	2046
	Albumin	*ALB*	*Gallus gallus*		71868	31	17	1258
	Tubulin alpha-4 chain (fragment)	*TBA4*	*Gallus gallus*		36483	22	26	969
	Heat shock cognate 71 kDa protein	*HSPA8*	*Gallus gallus*		71011	23	19	760
	Creatine kinase B-type	*CKB*	*Gallus gallus*		43129	17	25	735
	Acrosin	*ACR*	*Meleagris gallopavo*		38724	10	9	637
	Creatine kinase S-type (mitochondrial form)	*CKMT2*	*Gallus gallus*		47510	9	8	569
	Tubulin alpha-2 chain	*TBA2*	*Gallus gallus*		50450	16	16	552
	Voltage-dependent anion-selective channel protein 2	*VDAC2*	*Meleagris gallopavo*		30162	6	8	327
	EF-hand domain-containing family member C2	*EFHC2*	*Gallus gallus*		87461	3	3	146
	Outer dense fiber protein 2	*ODF2*	*Gallus gallus*		96467	3	3	132
	Glyceraldehyde-3-phosphate dehydrogenase	*GAPDH*	*Gallus gallus*		35909	2	4	107
	Glyceraldehyde-3-phosphate dehydrogenase	*GAPDH*	*Meleagris gallopavo*		25065	2	4	107
	ATP synthase subunit beta (mitochondrial form)	*ATP5F1B*	*Gallus gallus*		56650	2	2	87
	Heat shock protein HSP 90 kDa	*HSP90AA1*<br>*HSP90AB1*	*Gallus gallus*		84406<br>83717	2	1	77
	Elongation factor 1-alpha 1	*EEF1A*	*Gallus gallus*		50467	2	2	69
	Pyruvate kinase PKM	*PKM*	*Gallus gallus*		58434	2	2	63
**2**	Tubulin beta-3 chain	*TBB3*	*Gallus gallus*	51 kDa	50285	275	64	9253
	Tubulin beta-2 chain	*TBB2*	*Gallus gallus*		50377	1	53	52
	Tubulin beta-7 chain	*TBB7*	*Gallus gallus*		50095	204	55	5863
	Tubulin beta-5 chain	*TBB5*	*Gallus gallus*		50395	146	55	4734
	Tubulin beta-4 chain	*TBB4*	*Gallus gallus*		50844	151	30	4344
	Tubulin alpha-4 chain (fragemnt)	*TBA4*	*Gallus gallus*		36483	34	26	1516
	Tubulin alpha-5 chain	*TBA5*	*Gallus gallus*		50715	43	27	1299

	Tubulin alpha-2 chain	*TBA2*	*Gallus gallus*		50450	23	12	879
	ATP synthase subunit beta (mitochondrial form)	*ATP5F1B*	*Gallus gallus*	51 kDa	56650	8	14	321
	Phosphoglycerate kinase	*PGK*	*Gallus gallus*		45087	1	3	68
	Albumin	*ALB*	*Gallus gallus*		71868	5	4	186
	Acrosin	*ACR*	*Meleagris gallopavo*		38724	2	9	127
	Elongation factor 1-alpha 1	*EEF1A*	*Gallus gallus*		50467	2	2	62
	Cysteine protease ATG4B	*ATG4B*	*Gallus gallus*		45186	1		44
**3**	Tubulin beta-3 chain	*TBB3*	*Gallus gallus*		50285	156	57	5103
	Tubulin beta-2 chain	*TBB2*	*Gallus gallus*		50377	124	39	3524
	Tubulin beta-4 chain	*TBB4*	*Gallus gallus*		50844	93	52	2781
	Tubulin beta-5 chain	*TBB5*	*Gallus gallus*		50395	87	5	2738
	Creatine kinase B-type	*CKB*	*Gallus gallus*		43129	21		1150
	Tubulin alpha-4 chain (fragemnt)	*TBA4*	*Gallus gallus*		36483	18	26	871
	Albumin	*ALB*	*Gallus gallus*		71868	24	18	861
	Acrosin	*ACR*	*Meleagris gallopavo*		38724	11	15	651
	Tubulin alpha-2 chain	*TBA2*	*Gallus gallus*		50450	14	19	532
	Creatine kinase S-type (mitochondrial form)	*CKMT2*	*Gallus gallus*		47510	8	17	496
	ATP synthase subunit beta (mitochondrial form)	*ATP5F1B*	*Gallus gallus*		56650	14	24	401
	Voltage-dependent anion-selective channel protein 2	*VDAC2*	*Meleagris gallopavo*		30162	4	18	260
	Heat shock 70 kDa protein	*HSP70*	*Gallus gallus*	49 kDa	69936	9	12	247
	EF-hand domain-containing family member C2	*EFHC2*	*Gallus gallus*		87461	5	3	240
	Outer dense fiber protein 2	*ODF2*	*Gallus gallus*		96467	3	6	181
	Succinate dehydrogenase (mitochondrial form)	*SDHA*	*Gallus gallus*		74025	1	2	46
	Elongation factor 1-alpha 1	*EEF1A*	*Gallus gallus*		50467	2	2	70
	1-phosphatidylinositol 4,5-bisphosphate phosphodiesterase zeta-1	*PLCZ1*	*Gallus gallus*		73285	9	2	240
	Malate dehydrogenase (cytoplasmic form)	*MDH1*	*Gallus gallus*		36748	2	3	107
	Actin	*ACTA1*<br>*ACTA2*<br>*ACTB*<br>*ACTC*<br>*ACTG*<br>*ACTG2*<br>*ACT5*	*Gallus gallus*		42366<br>42367<br>42052<br>42334<br>42108<br>42249<br>42151	1	4	71
	Stress-70 protein (mitochondrial form)	*HSPA9*	*Gallus gallus*		73432	1	1	51

	Pyruvate kinase PKM	*PKM*	*Gallus gallus*	49 kDa	58434	1	2	48
	Cytochrome c	*CYC*	*Gallus gallus*<br>*Meleagris gallopavo*		11817<br>11817	1<br>1	12<br>12	45<br>45
**4**	Tubulin beta-3 chain	*TBB3*	*Gallus gallus*	43 kDa	50285	86	49	2577
	Albumin	*ALB*	*Gallus gallus*		71868	59	53	2460
	Creatine kinase B-type	*CKB*	*Gallus gallus*		43129	42	33	2208
	Tubulin beta-5 chain	*TBB5*	*Gallus gallus*		50395	58	22	1724
	Tubulin beta-4 chain	*TBB4*	*Gallus gallus*		50844	50	24	1650
	Tubulin alpha-1 chain	*TBA1*	*Gallus gallus*		46385	33	38	1175
	Creatine kinase S-type (mitochondrial form)	*CKMT2*	*Gallus gallus*		47510	13	26	691
	Tubulin alpha-4 chain (fragment)	*TBA4*	*Gallus gallus*		36483	10	22	563
	Acrosin	*ACR*	*Meleagris gallopavo*		38724	7	15	486
	Heat shock 70 kDa protein	*HSP70*	*Gallus gallus*		69936	9	11	230
	Voltage-dependent anion-selective channel protein 2	*VDAC2*	*Meleagris gallopavo*		30162	4	15	209
	Actin (cytoplasmic 2 form)	*ACTG1*	*Gallus gallus*		42108	3	9	159
	Aspartate aminotransferase (mitochondrial form)	*GOT2*	*Gallus gallus*		47496	3	6	147
	EF-hand domain-containing family member C2	*EFHC2*	*Gallus gallus*		87461	2	3	93
	Succinate dehydrogenase (mitochondrial form)	*SDHA*	*Gallus gallus*		74025	2	2	91
	ATP synthase subunit beta (mitochondrial form)	*ATP5F1B*	*Gallus gallus*		56650	2	5	86
	Phosphoglycerate kinase	*PGK*	*Gallus gallus*		45087	1	3	68
	Elongation factor 1-alpha 1	*EEF1A*	*Gallus gallus*		50467	2	2	66
	Stress-70 protein (mitochondrial form)	*HSPA9*	*Gallus gallus*		73432	1	1	51
	Pyruvate kinase PKM	*PKM*	*Gallus gallus*		58434	1	4	46
**5**	Creatine kinase B-type	*CKB*	*Gallus gallus*	40 kDa	43129	55	23	2758
	Albumin	*ALB*	*Gallus gallus*		71868	42	51	1592
	Creatine kinase S-type (mitochondrial form)	*CKMT2*	*Gallus gallus*		47510	39	30	1333
	Tubulin beta-3 chain	*TBB3*	*Gallus gallus*		50285	36	33	1005
	Tubulin beta-5 chain	*TBB5*	*Gallus gallus*		50395	25	19	693
	Tubulin beta-4 chain	*TBB4*	*Gallus gallus*		50844	24	32	631
	Aspartate aminotransferase (cytoplasmic form)	*GOT1*	*Gallus gallus*		46134	9	9	442
	Acrosin	*ACR*	*Meleagris gallopavo*		38724	5	15	328
	Voltage-dependent anion-selective channel protein 2	*VDAC2*	*Meleagris gallopavo*		30162	4	20	204
	Aspartate aminotransferase (mitochondrial form)	*GOT2*	*Gallus gallus*		47496	7	13	177
	Actin	*ACTA1*<br>*ACTA2*	*Gallus gallus*		42366<br>42367	2	4	110

		ACTB			42052			
		ACTC			42334			
		ACTG		40 kDa	42108			
		ACTG2			42249			
		ACT5			42151			
	Malate dehydrogenase (cytoplasmic form)	*MDH1*	*Gallus gallus*		36748	1	5	86
	Creatine kinase M-type	*CKM*	*Gallus gallus*		43529	1	4	82
	Succinate dehydrogenase (mitochondrial form)	*SDHA*	*Gallus gallus*		74025	1	1	46
**6**	Albumin	*ALB*	*Gallus gallus*		71868	58	47	2535
	Ovotransferrin	*TRFE*	*Gallus gallus*		79551	32	22	1388
	Tubulin beta-3 chain	*TBB3*	*Gallus gallus*		50285	27	37	780
	Heat shock cognate 71 kDa protein	*HSPA8*	*Gallus gallus*		71011	20	26	719
	Tubulin beta-5 chain	*TBB5*	*Gallus gallus*		50395	14	14	422
	Voltage-dependent anion-selective channel protein 2	*VDAC2*	*Meleagris gallopavo*		30162	8	31	410
	Tubulin beta-4 chain	*TBB4*	*Gallus gallus*		50844	14	41	365
	Acrosin	*ACR*	*Meleagris gallopavo*		38724	4	15	295
	Tubulin alpha-5 chain	*TBA5*	*Gallus gallus*		50715	6	12	273
	Stress-70 protein (mitochondrial form)	*HSPA9*	*Gallus gallus*		73432	9	10	272
	Ubiquitin-ribosomal protein eS31 fusion protein	*RPS27A*	*Gallus gallus*		18310	4	39	214
	Polyubiquitin-B	*UBB*	*Gallus gallus*	29 kDa	34348	4	13	214
	Creatine kinase S-type (mitochondrial form)	*CKMT2*	*Gallus gallus*		47510	3	5	180
	Creatine kinase B-type	*CKB*	*Gallus gallus*		43129	3	5	156
	Heat shock protein HSP 90 kDa	*HSP90AA1*	*Gallus gallus*		84406	5	6	143
		HSP90AB1			83717	5	6	143
	Malate dehydrogenase (cytoplasmic form)	*MDH1*	*Gallus gallus*		36748	2	14	123
	Elongation factor 1-alpha 1	*EEF1A*	*Gallus gallus*		50467	3	7	106
	ATP synthase subunit beta (mitochondrial form)	*ATP5F1B*	*Gallus gallus*		56650	3	8	105
	Succinate dehydrogenase (mitochondrial form)	*SDHA*	*Gallus gallus*		74025	1	2	99
	Glyceraldehyde-3-phosphate dehydrogenase	*GAPDH*	*Gallus gallus*		35909	2	4	91
	Glyceraldehyde-3-phosphate dehydrogenase	*GAPDH*	*Meleagris gallopavo*		25065	2	4	91
	Fatty acid synthase	*FASN*	*Gallus gallus*		277630	2	1	75
	Radixin	*RDX*	*Gallus gallus*		68626	2	1	72
	Cell cycle control protein 50A	*TMEM30A*	*Gallus gallus*		41821	2	2	72
	Elongation factor 2	*EEF2*	*Gallus gallus*		96343	1	2	69
	Protein-glutamine gamma-glutamyltransferase 2	*TGM2*	*Gallus gallus*	29 kDa	79118	1	2	68

	Probable cation-transporting ATPase 13A4	*ATP13A4*	*Gallus gallus*		135682	2	1	68
	Mitochondrial proton/calcium exchanger protein	*LETM1*	*Gallus gallus*		86422	1	1	54
	Gelsolin	*GSN*	*Gallus gallus*		86120	1	1	46
	Aspartate aminotransferase (cytoplasmic form)	*GOT1*	*Gallus gallus*		46134	1	3	46
**7**	Albumin	*ALB*	*Gallus gallus*		71868	72	42	2853
	Voltage-dependent anion-selective channel protein 2	*VDAC2*	*Meleagris gallopavo*		30162	21	42	1052
	Creatine kinase B-type	*CKB*	*Gallus gallus*		43129	18	28	1038
	Tubulin beta-3 chain	*TBB3*	*Gallus gallus*		50285	33	45	848
	Acrosin	*ACR*	*Meleagris gallopavo*		38724	12	20	722
	Tubulin beta-4 chain	*TBB4*	*Gallus gallus*		50844	22	43	523
	Tubulin beta-5 chain	*TBB5*	*Gallus gallus*		50395	19	19	503
	Creatine kinase S-type (mitochondrial form)	*CKMT2*	*Gallus gallus*		47510	7	14	412
	Ig lambda chain C region	*LAC*	*Gallus gallus*		11525	8	11	330
	Apolipoprotein A-I	*APOA1*	*Gallus gallus*		30661	10	15	301
	Tubulin alpha-4 chain (fragment)	*TBA4*	*Gallus gallus*		36483	6	21	301
	Actin	*ACTA1*<br>*ACTA2*<br>*ACTB*<br>*ACTC*<br>*ACTG*<br>*ACTG2*<br>*ACT5*	*Gallus gallus*	24 kDa	42366<br>42367<br>42052<br>42334<br>42108<br>42249<br>42151	4	9	217
	Phosphoglycerate kinase	*PGK*	*Gallus gallus*		45087	5	15	180
	ATP synthase subunit beta (mitochondrial form)	*ATP5F1B*	*Gallus gallus*		56650	6	13	171
	Phosphoglycerate mutase 1	*PGAM1*	*Gallus gallus*		29051	3	21	154
	Succinate dehydrogenase iron-sulfur subunit (mitochondrial form)	*SDHB*	*Gallus gallus*		33374	4	9	146
	Astacin-like metalloendopeptidase	*ASTL*	*Gallus gallus*		46929	3	3	132
	Tubulin alpha-2 chain	*TBA2*	*Gallus gallus*		50450	4	9	127
	Malate dehydrogenase (cytoplasmic form)	*MDH1*	*Gallus gallus*		36748	3	9	114
	Glyceraldehyde-3-phosphate dehydrogenase	*GAPDH*	*Gallus gallus*		35909	3	11	111
	Glyceraldehyde-3-phosphate dehydrogenase	*GAPDH*	*Meleagris gallopavo*		25065	3	11	111
	EF-hand domain-containing family member C2	*EFHC2*	*Gallus gallus*		87461	2	1	105
	Mitochondria-eating protein	*SPATA18*	*Gallus gallus*		54997	1	3	99
	Pyruvate kinase PKM	*PKM*	*Gallus gallus*		58434	2	6	92

	Outer dense fiber protein 2	*ODF2*	*Gallus gallus*	24 kDa	96467	1	2	92
	Heat shock cognate protein HSP 90-beta	*HSP90AB1*	*Gallus gallus*		83717	2	3	85
	Carbonic anhydrase 2	*CA2*	*Gallus gallus*		29388	1	2	83
	Succinate dehydrogenase (mitochondrial form)	*SDHA*	*Gallus gallus*		74025	1	2	83
	Ras-related protein Rab-10	*RAB10*	*Gallus gallus*		22763	2	5	82
	Creatine kinase M-type	*CKM*	*Gallus gallus*		43529	1	4	82
	Cytochrome b-c1 complex subunit Rieske (mitochondrial form)	*UQCRFS1*	*Gallus gallus*		29710	1	7	81
	L-lactate dehydrogenase B chain	*LDHB*	*Gallus gallus*		36694	1	3	74
	Elongation factor 1-alpha 1	*EEF1A*	*Gallus gallus*		50467	2	2	69
	Heat shock cognate 71 kDa protein	*HSPA8*	*Gallus gallus*		71011	1	3	68
	Probable cation-transporting ATPase 13A4	*ATP13A4*	*Gallus gallus*		135682	2	1	68
	Aspartate aminotransferase (cytoplasmic form)	*GOT1*	*Gallus gallus*		46134	1	5	67
	Elongation factor 2	*EEF2*	*Gallus gallus*		96343	1	2	72
	T-complex protein 1 subunit eta	*CCT7*	*Gallus gallus*		60881	1	4	66
	ATP-dependent RNA helicase DDX1	*DDX1*	*Gallus gallus*		83458	1	2	65
	Triosephosphate isomerase	*TPI1*	*Gallus gallus*		26832	2	6	80
	Ubiquitin-ribosomal protein eS31 fusion protein	*RPS27A*	*Gallus gallus*		18310	1	23	93
	Polyubiquitin-B	*UBB*	*Gallus gallus*		34348	1	7	93
	Glutathione S-transferase 3	*GST*	*Gallus gallus*		25282	2	10	60
	Peroxiredoxin-1	*PRDX1*	*Gallus gallus*		22529	2	5	65
	T-complex protein 1 subunit zeta	*CCT6*	*Gallus gallus*		58008	1	2	50
	Fructose-bisphosphate aldolase C	*ALDOC*	*Gallus gallus*		14543	1	4	49
	Rho-related GTP-binding protein RhoC	*RHOC*	*Gallus gallus*		22301	1	8	44
**8**	Albumin	*ALB*	*Gallus gallus*	18 kDa	71868	95	76	4252
	Tubulin beta-3 chain	*TBB3*	*Gallus gallus*		50285	37	50	1241
	Acrosin	*ACR*	*Meleagris gallopavo*		38724	17	26	986
	Tubulin beta-5 chain	*TBB5*	*Gallus gallus*		50395	25	22	833
	Creatine kinase B-type	*CKB*	*Gallus gallus*		43129	15	34	818
	Voltage-dependent anion-selective channel protein 2	*VDAC2*	*Meleagris gallopavo*		30162	16	46	807
	Tubulin beta-4 chain	*TBB4*	*Gallus gallus*		50844	27	28	791
	Creatine kinase S-type (mitochondrial form)	*CKMT2*	*Gallus gallus*		47510	9	19	580
	Tubulin alpha-4 chain (fragment)	*TBA4*	*Gallus gallus*		36483	11	22	553
	Astacin-like metalloendopeptidase	*ASTL*	*Gallus gallus*		46929	6	11	225
	Malate dehydrogenase (cytoplasmic form)	*MDH1*	*Gallus gallus*		36748	4	5	171

Ig lambda chain C region	*LAC*	*Gallus gallus*		11525	5	11	171
Heat shock cognate 71 kDa protein	*HSPA8*	*Gallus gallus*		71011	4	11	150
Aspartate aminotransferase (mitochondrial form)	*GOT2*	*Gallus gallus*		47496	3	5	149
Ubiquitin-ribosomal protein eS31 fusion protein	*RPS27A*	*Gallus gallus*		18310	3	44	143
Polyubiquitin-B	*UBB*	*Gallus gallus*		34348	3	14	143
Retinol-binding protein 4	*RBP4*	*Gallus gallus*		22843	3	21	134
Cytochrome b-c1 complex subunit Rieske (mitochondrial form)	*UQCRFS1*	*Gallus gallus*		29710	2	13	125
Ras-related protein Rap-1b	*RAP1B*	*Gallus gallus*		21040	4	13	123
Ovotransferrin	*TRFE*	*Gallus gallus*		79551	3	6	122
Actin	*ACTA1*<br>*ACTA2*<br>*ACTB*<br>*ACTC*<br>*ACTG*<br>*ACTG2*<br>*ACT5*	*Gallus gallus*	18 kDa	42366<br>42367<br>42052<br>42334<br>42108<br>42249<br>42151	2	4	122
Pyruvate kinase PKM	*PKM*	*Gallus gallus*		58434	3	9	111
Cilia- and flagella-associated protein 20	*CFAP20*	*Gallus gallus*		22905	3	15	106
Heat shock protein HSP 90 kDa	*HSP90AA1*<br>*HSP90AB1*	*Gallus gallus*		84406<br>83717	2<br>2	1<br>1	103<br>103
Transthyretin	*TTR*	*Gallus gallus*		16356	2	8	102
Cell division control protein 42 homolog	*CDC42*	*Gallus gallus*		21601	2	10	82
1-phosphatidylinositol 4,5-bisphosphate phosphodiesterase zeta-1	*PLCZ1*	*Gallus gallus*		73285	1	2	80
Serine/threonine-protein phosphatase PP1-beta catalytic subunit	*PPP1CB*	*Gallus gallus*		37961	1	3	78
GTPase HRas<br>GTPase NRas	*HRAS*<br>*NRAS*	*Gallus gallus*		21694<br>21550	1	6	74
Protein/nucleic acid deglycase DJ-1	*PARK7*	*Gallus gallus*		20159	1	7	69
Phosphoglycerate kinase	*PGK*	*Gallus gallus*		45087	1	4	61
Platelet-activating factor acetylhydrolase IB subunit alpha2	*PAFAH1B2*	*Gallus gallus*		25665	2	3	60
Glyceraldehyde-3-phosphate dehydrogenase	*GAPDH*	*Gallus gallus*		35909	1	7	58
Glyceraldehyde-3-phosphate dehydrogenase	*GAPDH*	*Meleagris gallopavo*		25065	1	7	58

	Aspartate aminotransferase (cytoplasmic form)	*GOT1*	*Gallus gallus*	18 kDa	46134	1	3	58
	Peroxiredoxin-1	*PRDX1*	*Gallus gallus*		22529	1	5	57
	Apolipoprotein A-I	*APOA1*	*Gallus gallus*		30661	1	4	56
	Succinate dehydrogenase (mitochondrial form)	*SDHA*	*Gallus gallus*		74025	1	4	53
	Ferritin heavy chain	*FTH*	*Gallus gallus*		21249	1	6	47
**9**	Albumin	*ALB*	*Gallus gallus*	15 kDa	71868	70	62	3019
	Tubulin beta-3 chain	*TBB3*	*Gallus gallus*		50285	55	49	1701
	Creatine kinase B-type	*CKB*	*Gallus gallus*		43129	21	35	1121
	Tubulin beta-5 chain	*TBB5*	*Gallus gallus*		50395	29	19	992
	Acrosin	*ACR*	*Meleagris gallopavo*		38724	16	26	982
	Tubulin beta-4 chain	*TBB4*	*Gallus gallus*		50844	29	20	889
	Creatine kinase S-type (mitochondrial form)	*CKMT2*	*Gallus gallus*		47510	11	23	758
	Voltage-dependent anion-selective channel protein 2	*VDAC2*	*Meleagris gallopavo*		30162	14	52	755
	Tubulin alpha-4 chain (fragment)	*TBA4*	*Gallus gallus*		36483	9	22	465
	Cytochrome b-c1 complex subunit Rieske (mitochondrial form)	*UQCRFS1*	*Gallus gallus*		29710	6	13	359
	Cilia- and flagella-associated protein 20	*CFAP20*	*Gallus gallus*		22905	9	44	307
	Ubiquitin-ribosomal protein eS31 fusion protein	*RPS27A*	*Gallus gallus*		18310	5	63	255
	Polyubiquitin-B	*UBB*	*Gallus gallus*		34348	5	20	255
	Ras-related protein Rap-1b	*RAP1B*	*Gallus gallus*		21040	7	13	226
	Ferritin heavy chain	*FTH*	*Gallus gallus*		21249	7	19	209
	Succinate dehydrogenase (mitochondrial form)	*SDHA*	*Gallus gallus*		74025	5	13	194
	Retinol-binding protein 4	*RBP4*	*Gallus gallus*		22843	3	16	176
	Ig lambda chain C region	*LAC*	*Gallus gallus*		11525	5	11	153
	Astacin-like metalloendopeptidase	*ASTL*	*Gallus gallus*		46929	3	5	142
	Transthyretin	*TTR*	*Gallus gallus*		16356	2	8	126
	Lysozyme G	*LYG*	*Gallus gallus*		23565	3	8	125
	Ovotransferrin	*TRFE*	*Gallus gallus*		79551	2	1	102
	Zona pellucida-binding protein 1	*ZPBP1*	*Gallus gallus*		36910	4	6	98
	T-complex protein 1 subunit theta	*CCT8*	*Gallus gallus*		60017	2	6	95
	Apolipoprotein A-I	*APOA1*	*Gallus gallus*		30661	3	9	93
	Fatty acid-binding protein	*FABP7*	*Gallus gallus*		15031	1	16	86
	Cell division control protein 42 homolog	*CDC42*	*Gallus gallus*		21601	2	10	84
	Calmodulin	*CALM*<br>*CCM1*	*Gallus gallus*		16827<br>16990	1	11	82

	Creatine kinase M-type	*CKM*	*Gallus gallus*	15 kDa	43529	1	4	82
	Serine/threonine-protein phosphatase PP1-beta catalytic subunit	*PPP1CB*	*Gallus gallus*		37961	1	3	79
	Actin	*ACTA1*<br>*ACTA2*<br>*ACTB*<br>*ACTC*<br>*ACTG*<br>*ACTG2*<br>*ACT5*	*Gallus gallus*		42366<br>42367<br>42052<br>42334<br>42108<br>42249<br>42151	2	4	78
	GTPase HRas<br>GTPase NRas	*HRAS*<br>*NRAS*	*Gallus gallus*		21694<br>21550	1	6	61
	Malate dehydrogenase (cytoplasmic form)	*MDH1*	*Gallus gallus*		36748	1	9	54
	Solute carrier family 2, facilitated glucose transporter member 3	*SLC2A3*	*Gallus gallus*		54539	1	2	54
	Aspartate aminotransferase (mitochondrial form)	*GOT2*	*Gallus gallus*		47496	1	2	51
	Platelet-activating factor acetylhydrolase IB subunit alpha2	*PAFAH1B2*	*Gallus gallus*		25665	1	3	47
**10**	Tubulin beta chain	*TBB3*	*Gallus gallus*	13 kDa	50285	303	69	9548
		TBB2			50377	285	54	9083
		TBB7			50095	212	57	6075
		TBB4			50844	134	30	4023
		TBB5			50395	119	24	3740
	Tubulin alpha chain	*TBA5*	*Gallus gallus*		50715	45	36	1526
		TBA4			36483	30	30	1351
	ATP synthase subunit beta (mitochondrial form)	*ATP5F1B*	*Gallus gallus*		56650	38	47	1306
	Creatine kinase B-type	*CKB*	*Gallus gallus*		43129	22	31	1172
	Alpha-enolase	*ENO1*	*Gallus gallus*		47617	19	30	1039
	Tubulin alpha-2 chain	*TBA2*	*Gallus gallus*		50450	23	17	873
	Heat shock cognate 71 kDa protein	*HSPA8*	*Gallus gallus*		71011	22	27	793
	Creatine kinase S-type (mitochondrial form)	*CKMT2*	*Gallus gallus*		47510	12	23	668
	Heat shock 70 kDa protein	*HSP70*	*Gallus gallus*		69936	13	14	364
	Acrosin	*ACR*	*Meleagris gallopavo*		38724	6	15	359
	Voltage-dependent anion-selective channel protein 2	*VDAC2*	*Meleagris gallopavo*		30162	5	15	230
	Outer dense fiber protein 2	*ODF2*	*Gallus gallus*		96467	4	2	177

Succinate dehydrogenase (mitochondrial form)	*SDHA*	*Gallus gallus*	13 kDa	74025	2	2	160
Actin	*ACTA1*<br>*ACTA2*<br>*ACTB*<br>*ACTC*<br>*ACTG*<br>*ACTG2*<br>*ACT5*	*Gallus gallus*		42366<br>42367<br>42052<br>42334<br>42108<br>42249<br>42151	3	4	147
EF-hand domain-containing family member C2	*EFHC2*	*Gallus gallus*		87461	2	3	124
Ubiquitin-ribosomal protein eS31 fusion protein	*RPS27A*	*Gallus gallus*		18310	2	47	109
Polyubiquitin-B	*UBB*	*Gallus gallus*		34348	2	15	109
Pyruvate kinase PKM	*PKM*	*Gallus gallus*		58434	3	9	105
Elongation factor 1-alpha 1	*EEF1A*	*Gallus gallus*		50467	3	7	102
Na^+/H^+ exchange regulatory cofactor NHE-RF1	*NHERF1*	*Gallus gallus*		36011	1	6	92
Malate dehydrogenase (cytoplasmic form)	*MDH1*	*Gallus gallus*		36748	2	5	76
Stress-70 protein (mitochondrial form)	*HSPA9*	*Gallus gallus*		73432	1	1	57
Phosphoglycerate kinase	*PGK*	*Gallus gallus*		45087	1	3	54
Triosephosphate isomerase	*TPI1*	*Gallus gallus*		26832	1		48
Radixin	*RDX*	*Gallus gallus*		68626	1	1	47
